# Transcriptomic analysis of the interaction between *Helianthus annuus *and its obligate parasite *Plasmopara halstedii *shows single nucleotide polymorphisms in CRN sequences

**DOI:** 10.1186/1471-2164-12-498

**Published:** 2011-10-11

**Authors:** Falah As-sadi, Sébastien Carrere, Quentin Gascuel, Thibaut Hourlier, David Rengel, Marie-Christine Le Paslier, Amandine Bordat, Marie-Claude Boniface, Dominique Brunel, Jérôme Gouzy, Laurence Godiard, Patrick Vincourt

**Affiliations:** 1 INRA, Laboratoire des Interactions Plantes-Microorganismes (LIPM), UMR441, F-31326 Castanet-Tolosan, France. CNRS, Laboratoire des Interactions Plantes-Microorganismes (LIPM), UMR2594, F-31326 Castanet-Tolosan, France; 2 Etude de Polymorphisme des Génomes Végétaux (INRA), CEA, Institut de Génomique, Centre National de Génotypage, 2 rue Gaston Crémieux CP 5721, F-91057 Évry Cedex, France

## Abstract

**Background:**

Downy mildew in sunflowers (*Helianthus annuus *L.) is caused by the oomycete *Plasmopara halstedii *(Farl.) Berlese et de Toni. Despite efforts by the international community to breed mildew-resistant varieties, downy mildew remains a major threat to the sunflower crop. Very few genomic, genetic and molecular resources are currently available to study this pathogen. Using a 454 sequencing method, expressed sequence tags (EST) during the interaction between *H. annuus *and *P. halstedii *have been generated and a search was performed for sites in putative effectors to show polymorphisms between the different races of *P. halstedii*.

**Results:**

A 454 pyrosequencing run of two infected sunflower samples (inbred lines XRQ and PSC8 infected with race 710 of *P. halstedii*, which exhibit incompatible and compatible interactions, respectively) generated 113,720 and 172,107 useable reads. From these reads, 44,948 contigs and singletons have been produced. A bioinformatic portal, HP, was specifically created for in-depth analysis of these clusters. Using *in silico *filtering, 405 clusters were defined as being specific to oomycetes, and 172 were defined as non-specific oomycete clusters. A subset of these two categories was checked using PCR amplification, and 86% of the tested clusters were validated. Twenty putative RXLR and CRN effectors were detected using PSI-BLAST. Using corresponding sequences from four races (100, 304, 703 and 710), 22 SNPs were detected, providing new information on pathogen polymorphisms.

**Conclusions:**

This study identified a large number of genes that are expressed during *H. annuus/P. halstedii *compatible or incompatible interactions. It also reveals, for the first time, that an infection mechanism exists in *P. halstedii *similar to that in other oomycetes associated with the presence of putative RXLR and CRN effectors. SNPs discovered in CRN effector sequences were used to determine the genetic distances between the four races of *P. halstedii*. This work therefore provides valuable tools for further discoveries regarding the *H. annuus/P. halstedii *pathosystem.

## Background

Downy mildew in sunflowers (*Helianthus annuus *L.) is caused by the oomycete *Plasmopara halstedii *(Farl.) Berlese et de Toni. Both the host plant and the pathogen species originated in North America, where co-evolution has taken place [1]. As the result of the fast evolution of the pathogen and despite considerable efforts by public research and seed companies, downy mildew remains a major risk for the crop, as new races of the pathogen are bypassing the resistance of sunflower hybrids [2], which is generally based on race-specific *Pl *genes [3-6]. One of the strategies used to enhance the durability of disease resistance to downy mildew in the field consists of identifying quantitative resistance loci in plants [7] and improving the knowledge about the genetic variability of the pathogen to make molecular tools available that will assist in genotyping new *P. halstedii *isolates. Previous studies have already been conducted at the molecular level to analyze the interaction between *H. annuus *and *P. halstedii *[4-6,8,9]. Downy mildew isolates have been collected from diseased plants in the field and designated as races based on their divergent virulence profiles in a set of differential hosts that carry different *Pl *resistance genes [2]. Fourteen different reference races of this pathogen have now been characterized in France, nine of which emerged in the last ten years [10]. Using a combination of SNP markers [11], Delmotte et al. [10] analyzed 24 individual isolates covering all 14 races that are found in France. Using these data, they observed a strong correlation between genetic and phenotypic structure, indicating that the 14 races fall into three distinct groups. Each of these genetically differentiated groups included one of the main races found in France: 100, 703, and 710 [2]. However, the genetic structure evidence might only reflect the neutral genetic structure of French *P. halstedii *populations because the SNP markers used do not provide any relevant functional information with respect to pathogenicity profiles.

*P. halstedii *is an oomycete from the Peronosporaceae family. Oomycetes form a group which is distinct from fungi [12,13] and include many plant pathogens, such as *Phytophthora *and downy mildews (*Bremia, Peronospora, Plasmopara*). The pathosystems *Hyaloperonospora arabidopsidis/Arabidopsis thaliana *and *Bremia lactucae/Lactuca sativa *are well-studied model systems for downy mildews (see [14] and [15] for review). Downy mildews are obligate biotrophs and therefore require living hosts to survive. Using infection structures, such as haustoria, the pathogen draws nutrients from its host and releases enzymes and effectors into the host's cells.

Effectors are defined as key elements of pathogenicity that have been shown modulating the host's defense system and enabling tissue colonization in other model pathosystems [16-18], but not in the Helianthus annuus * *Plasmopara halstedii *pathosystem up to now. In oomycetes, two classes of cytoplasmic effectors have been characterized, RXLR and CRN (for crinkling and necrosis) [19,20]. The RXLR-dEER motif of the RXLR protein family was discovered by comparing the protein sequences of AVR1b, AVR3a, ATR1 and ATR13 [20-22]. CRN1 and CRN2 are two cell-death-inducing proteins that cause crinkling and necrosis phenotypes in the leaves of infected plants [23].

Bouzidi et al. [8] used a genomics approach to identify genes involved in the *H. annuus/P. halstedii *interaction. They employed a subtractive hybridization method (SSH) in sunflower seedlings infected by *P. halstedii*. A total of 145 ESTs were identified as specific to the oomycete, but no effector was highlighted.

The advent of next-generation sequencing methods with reduced costs and higher throughput has encouraged the generation of more comprehensive and in-depth studies for a wider range of organisms and transcriptomes [24-26]. One of these methods that makes it possible to generate valuable information for species with high economic interest but limited genomic resources is 454 pyrosequencing technology [27]. In addition, 454 sequencing allows the identification of allelic variations and constitution of haplotypes [28].

In the context of sustainable agriculture, management of durable genetic resistance and minimization of selective pressure on pathogens are key objectives. Enriching the genomic resources available for exploring the interaction between *H. annuus *and *P. halstedii *is crucial for research on *P. halstedii*, especially with respect to discovering the effectors involved in its pathogenicity. In this study, a 454 FLX pyrosequencing of cDNAs from *H. annuus *seedlings infected by *P. halstedii *was performed to produce sequences expressed by either organism in the frame of their interaction. The resulting assembly was searched for effectors, such as RXLR and CRN; the polymorphisms of these effectors between the four races of *P. halstedii *were used as markers for re-evaluating their genetic relationships.

## Results and Discussion

### 454 pyrosequencing and assembly of HP clusters

Two sunflower lines that are susceptible (PSC8) or resistant (XRQ) to infection by *P. halstedii *race 710 were analyzed. The PSC8 samples infected with *P. halstedii *race 710 (a compatible interaction) generated 251,126 reads (with an average length of 176 bp and a median length of 161 bp), while the infected XRQ samples (an incompatible interaction) generated 161,526 reads (with an average length of 184 bp and a median length of 179 bp). After trimming and cleaning procedures, 172,107 (XRQ) and 113,720 (PSC8) useable reads were obtained.

These reads were pooled with 134,030 *H. annuus *EST and mRNA sequences and 145 *P. halstedii *EST and mRNA sequences that were available in GenBank (January 2009) to assemble clusters. This clustering produced 44,948 contigs and singletons. The HP database produced is available at http://www.heliagene.org/HP. Of these, 25,381 HP clusters are considered new because they could not be assembled with any publically available *H. annuus *or *P. halstedii *ESTs. The clusters obtained were annotated as HPXXXXX (where × represents a digit), and for simplicity, they will be designated as HP clusters hereafter.

**Figure 1:**
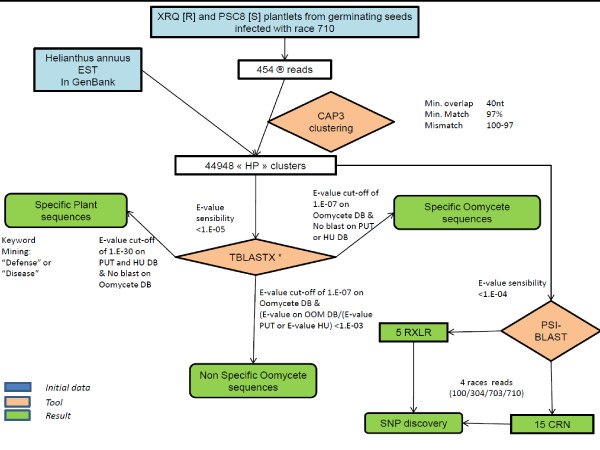
**Workflow of the study analysis**. HU = Heliagene database (DB), * = performed on Heliagene DB, GenBank DB, PUT DB, SwissProt DB, TAIR DB and a local oomycete database (OOM).

Information sequence similarities for each cluster were collected from the Heliagene (a sunflower database, http://www.heliagene.org; see Methods for more information), GenBank (NCBI), TAIR, PUT (PlantGDB-assembled Unique Transcripts [29]), InterPro, and SwissProt databases and were incorporated in the HP database. An oomycete database (OOM) was created from all of the oomycete sequences available in GenBank (February 2010) and was thereafter updated to include the *Hyaloperonospora arabidopsidis *sequences that were made available to the scientific community in December 2010 ([30], see Methods for more information). This database was used to search for similarities with oomycete sequences. This information was also incorporated in the HP database. Figure 1 describes the analysis workflow used in this study.

### *In silico *identification of *P. halstedii *sequences

The HP clusters originated from cDNAs of infected sunflower samples and could therefore correspond to either *H. annuus *or *P. halstedii*. To identify putative *Plasmopara *sequences, an *in silico *selection procedure was applied, based on TBLASTX results that were obtained from different databases (PUT, Heliagene and OOM) [31].

**Figure 2:**
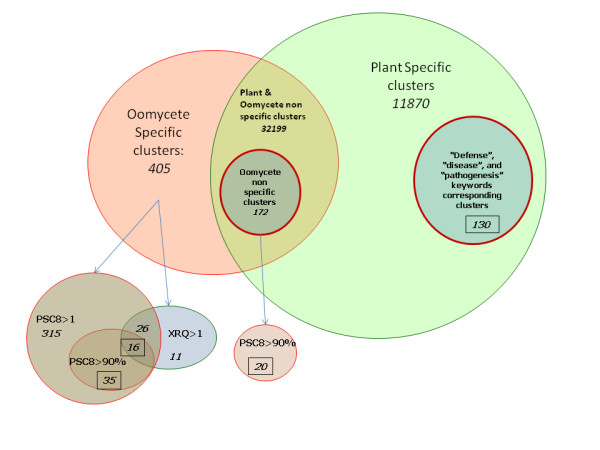
**Venn diagram sorting clusters between *Plasmopara *and *Helianthus *putative sequences**. In *italics*: numbers in the subclass. Surrounded by a square: a subset of clusters that were tested by PCR amplification to check their belonging subclass. PSC8 > 90%: clusters formed mainly by reads originated from PSC8 sample (compatible reaction). XRQ > 1: clusters having at least one read originated from XRQ sample (incompatible interaction). PSC8 > 1: clusters having at least one read originated from PSC8 sample.

First, using TBLASTX searches with (i) an expect value (E-value) lower than 1e-07 against the OOM database and (ii) an E-value greater than 1e-04 against the PUT or Heliagene databases, which indicated poor match with plant sequences, 405 HP clusters were defined as specific oomycete sequences (Figure 2, Additional files 1 &2). Among these, 350 clusters were found to be specific to the infected, susceptible sunflower line, PSC8, while 11 clusters were specific to the infected, resistant sunflower line, XRQ. A total of 42 clusters were found to be common to both samples. Among the 405 candidate oomycete clusters, 51 clusters were highly represented in the infected PSC8 line (corresponding to more than 90% of the reads for the clusters that generated at least 10 reads), whereas none of the clusters were highly represented in XRQ. This can be explained by the resistant genetic background of the XRQ line with respect to race 710, which results in very little multiplication of *P. halstedii *in the tissues of these plants in contrast to the susceptible PSC8 plants. The high level of oomycete multiplication in infected PSC8 plants was expected given the susceptibility of PSC8 to the race 710, and was confirmed based on the large amount of *P. halstedii *ribosomal RNA in the total RNA in the PSC8 sample compared to the total RNA in the sample from the infected XRQ resistant line (data not shown).

Next, as some HP candidates could belong to either *H. annuus *or *P. halstedii *due to the *in silico *(TBLASTX) proximity of the two species, a class of "non-specific oomycete" clusters was selected using two TBLASTX criteria: (i) only HP clusters with an E-value < 1e-07 against the OOM database were considered, and (ii) among those, only the HP clusters for which E-values against the OOM database were 1,000 times smaller than the E-values against the PUT or Heliagene databases were retained. This selection led to the identification of 172 HP clusters as "non-specific oomycete" sequences that are expressed by the pathogen during interactions with the plant (Figure 2, Additional files 3 &4). Of these, 20 clusters were selected for validation based on the high proportion of reads (greater than 90%) in the PSC8-infected sample.

### Validation of the *in silico*-predicted oomycete sequences by PCR amplification with genomic DNA

**Figure 3:**
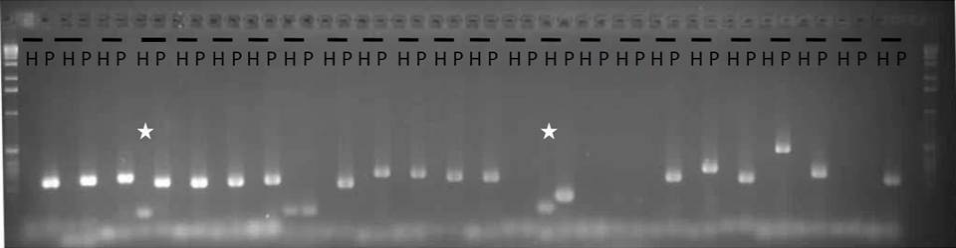
**PCR amplification of a subset of the 49 predicted oomycete sequences**. PCR amplification was performed with *H.annuus *PSC8 DNA (H) and *P. halstedii *race 710 DNA (P) with 24 primer couples, and loaded on an agarose gel (1.5%) stained with ethidium bromide. Ladder used was 1Kb (BioLabs). The star indicates an amplification on both H and P but with the H amplified band size smaller than that of P. The amplified HP represented are: HP003248, HP000331, HP001113, HP000314, HP003279, HP003590, HP003584, HP000639, HP003940, HP001561, HP001957, HP000353, HP001963, HP001040, HP001711, HP000298, HP003084, HP003006, HP000301, HP001391, HP000977, HP001858, HP002621, HP002636. The predicted amplified band sizes are indicated in Additional file 5.

Fifty-two sequences that were highly expressed in PSC8 (Additional file 5) were selected to check the accuracy of the *in silico *filtering. Thirty-five of these were specific oomycete clusters, while the remaining 17 sequences belonged to the non-specific oomycete category. They were tested by PCR amplification using *H. annuus *(inbred line XRQ) and *P. halstedii *(race 710) genomic DNA. A total of 39 amplifications produced a unique band when tested in *P. halstedii *DNA. Seven amplifications produced a band when using either *H. annuus *(H) or *P. halstedii *(P) DNA. No amplification was obtained with the last six clusters for either type of genomic DNA. Importantly, none of the 52 sequences produced amplified bands only when tested with *H. annuus *DNA, thus validating the *in silico *filtering process which was used in this study. Similar amplification patterns were obtained with PSC8 and race 710 genomic DNA. Regarding the six clusters that amplified both plant and oomycete genomic DNA, the band amplified from *P. halstedii *DNA exhibited the expected size, while the bands amplified using *H. annuus *DNA presented a lower molecular size (indicated by a star in Figure 3), suggesting that they are likely due to non-specific amplification, which can be caused by a homologous but shorter sequence in Helianthus or low-specificity primers. Due to the approximately 85% correct predictions and the lack of any amplifications being obtained only with *Helianthus *DNA, the *in silico *filtering method was considered as providing reliable results, and it was therefore assumed that a large majority of clusters were accurately allocated to their respective categories.

### Biological features of the *P. halstedii *predicted sequences

The 405 oomycete-specific clusters and the 20 non-specific oomycete clusters that were confirmed by PCR to be of *P. halstedii *origin were examined to describe the biological features of the potential new *P. halstedii *sequences. Among these 425 clusters, only 36 (8.5%) corresponded to *P. halstedii *sequences that were already present in the GenBank database, indicating that this study provides 389 potentially new *P. halstedii *genes that are expressed during interaction with sunflowers, increasing the number of sequences present in the database by at least 3 fold [8]. Of these 36 previously identified *P. halstedii *sequences, nine clusters correspond to ribosomal protein gene sequences (HP000627, HP001039, HP002564, HP003353, HP016471, HP018679, HP033486, HP034141, and HP034474); two encode a protein with an InterPro NAC motif (for nascent polypeptide-associated complex) (HP000902 and HP030154); one contained a putative WD40 domain; one was similar to a glucose transporter; one was similar to F1-ATP synthase; and one had an NADPH oxidoreductase domain. The remaining 21 clusters encoded unknown *P. halstedii *proteins and showed no significant similarities to other organisms (Additional files 1 and 3). Most of the 389 new *P. halstedii *sequences encoded putative proteins with unknown functions, and only 32 of the predicted proteins had an InterPro motif. These new *P. halstedii *cluster sequences presented strong TBLASTX E-values associated with different oomycete sequences present in the OOM database. A total of 310 clusters (73%) exhibited highest TBLASTX homology to seven *Phytophthora *species, with the most represented being *P. infestans *(27% of the hits) and *P. capsici *(27%). The other top similarity scores were found with *Hyaloperonospora arabidopsidis *(16.5% of the hits) and, to a much lesser extent, with *Phytophthora parasitica *(7%), *P. sojae *(6%), *P. brassicae *(4%) and *Pythium ultimum *(1.6%). No significant similarity was found with *Aphanomyces euteiches *sequences. These proportions are expected to be partly biased by the representation of the different oomycete species in the OOM database that was built (see Material and Methods) but likely also correspond to the phylogenetic relationships between the different oomycetes. Surprisingly, fewer hits were found against *Hyaloperonospora arabidopsidis*, which is considered as a phylogenetically close relative to *P. halstedii *[32]. Because *P. halstedii *is a member of the Peronosporaceae, it was not surprising to find that it is more closely related to Phytophthora species than to *P. ultimum *or *A. euteiches *(Saprolegniales). Phytophthora species are phylogenetically close to downy mildews, such as *P. halstedii *and *Hyaloperonospora arabidopsidis *[32]; both exhibit specialized infection structures called haustoria, and their genomes encode RXLR-EER type effectors (in contrast to *P. ultimum *and *A. euteiches*) ([33] and this study).

### Searching for *H. annuus *sequences expressed during infection by *P. halstedii*

Using a TBLASTX analysis with a cut-off E-value of 1e-30 against the PUT and Heliagene databases and no match to the OOM database, 12,000 HP clusters were predicted to be of plant origin. Among these, searches were carried out using the keywords "defense", "disease" and "pathogenesis" within the InterPro and GO fields of the TBLASTX results against different databases (PUT, Heliagene and SwissProt) and the InterPro Scan results. A total of 130 clusters was obtained (Figure 2, Additional file 6), and a total of 74 of those was tested to determine their host origin by PCR amplification using sunflower and downy mildew genomic DNA. Only 30% of the clusters were found to be specific to *H. annuus *(XRQ genomic DNA) (Additional file 7). Of these, eight putative NBS-LRRs (HP009300, HP009882, HP010230, HP020625, HP021629, HP022037, HP022395 and HP027120) were detected as well as an EDS-1 (for Enhanced Disease Susceptibility, HP016054), EDS-5 (HP021975) and EIN2 (for Ethylene Insensitivity, HP004696), while 40.5% of the clusters led to an amplified band of the same size using both *P. halstedii *(race 710) and *H. annuus *DNA. These results indicate that the filter requiring no match to the OOM database was insufficiently selective, likely due to the lack of *Plasmopara *sequences in the OOM database.

### Searching for *P. halstedii *putative effector sequences using PSI-BLAST

Recently, many studies have shown a vast repertoire of cytoplasmic and apoplastic effector proteins in oomycetes. Within the class of cytoplasmic effectors, the RXLR and Crinkler (CRN) families have been especially well studied [19,20]. RXLR and CRN proteins are secreted in haustoria and translocated into host cells to modulate host defenses and enable pathogenicity [34]. In addition to a signal peptide, RXLR proteins exhibit a characteristic RXLR amino acid motif that is sometimes associated with a -dEER motif, while CRN proteins show a characteristic LXLFLAK motif. The OOM database was searched for similarity to known oomycete effectors using the PSI-TBLASTN method (with an E-value cutoff of < 1e-04) [35]. All of the RXLR and CRN sequences available in GenBank were used to construct two corresponding matrices for the PSI-TBLASTN search. A total of 15 putative CRN clusters and five putative RXLR clusters were found in the HP database. These relatively short clusters obtained from cDNA that came from plants infected by race 710 were elongated using other cDNA sequences obtained by germinating spore materials from different races (see Methods and unpublished data from F. Delmotte et al.). The elongated, generated clusters are described in Additional files 8 &9.

**Figure 4:**
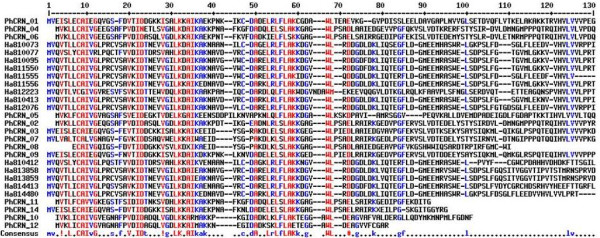
**Multalin alignment of the N-termini of 13 *P.halstedii *(PhCRN) and 8 *H.arabidopsidis *(HaCRN) CRN putative effectors**. PhCRN proteins were predicted by FrameDP [46] and for the alignment, first methionine was considered as the start of the protein. PhCRN_13 and_15, too short, were excluded. The predicted proteins for PhCRN_10 to_14 were added even their FrameDP predicted peptides were shorter than 130 amino acids. The last lane consensus indicates the highly conserved amino acids in red (> 90% identity) and in blue, amino acids conserved in more than 50% of the aligned sequences.

• **RXLR putative sequences: **The five putative *P. halstedii *RXLR proteins detected as being homologous to oomycete effectors by PSI-TBLASTN included a predicted signal peptide and are therefore likely secreted along with the EER motif. The following RXLR motives were found: RLLI (PhRXLR_02 and _03), RLLR (PhRXLR_05), or a putative motif (RKLQ in PhRXLR_01 or RALT in PhRXLR_04). The encoded predicted peptide sequences (Additional file 10) did not show any significant homology with known peptide sequences due in part to the large variability of RXLR sequences but also because they are short (70 to 140 amino acids), with the exception of PhRXLR_04 (334 amino acids).

• **CRN putative sequences: **Among the putative *P. halstedii *CRN sequences, 7 included the characteristic LXLFLAK CRN translocation motif 50 amino acids after the first methionine [33], and the eight others exhibited a close variant motif or LXLSLAK. Only 4 of these sequences presented a predicted signal peptide, but previous studies have made similar observations for CRN [36]. The Multalin [37] alignment of 13 of the PhCRN sequences showed a conserved N-terminal region of approximately 90 amino acids. In 8 out of 9 of the predicted peptides longer than 130 amino acids, a conserved HVLV(L/V)VP motif (at 120 amino acids) followed by variable C-terminal regions was found (Figure 4). This organization is reminiscent of CRN proteins from Phytophthora [38]. Alignment with 8 CRN proteins from *Hyaloperonospora arabidopsidis *(https://www.vbi.vt.edu/) showed a high degree of conservation of the first 90 amino acids between the two oomycete species, with 23 conserved (> 90% identity) and 20 conservative (> 50% identity) amino acid residues being observed, likely related to their close phylogenetic relationship (Figure 4) [32]. All of the PhCRN predicted peptide sequences exhibited TBLASTN hits with the OOM database of predicted gene models, which includes data on *H. arabidopsidis*. Ten out of 15 of these PhCRN sequences showed the best hit against *Phytophthora infestans*; the remaining sequences best matched *Phytophthora capsici *(Additional file 11). This result suggests that *P. halstedii *is closer to *Phytophthora *species than to *H. arabidopsidis *based on their CRN profiles. This provides new insight with respect to the relationship between the pathogenicity profiles of the oomycetes, which was not anticipated according to [32]. Most of the predicted sequences exhibited the best hit against uncharacterized proteins from *Phytophthora *sp. and did not give any clue regarding their putative function. Intriguingly, PhCRN_04, _06 and _11 include an InterPro domain, IPR002575, which corresponds to an aminoglycoside phosphotransferase domain that is typically found in bacterial genes and confers antibiotic resistance by phosphorylation [39]. As none of the predicted PhRXLR effector peptide sequences showed a hit against the same database, this suggests less specificity in PhCRN effectors than in RXLR effectors.

Our data indicate, for the first time, that *P. halstedii *exhibits the same kind of CRN and RXLR cytoplasmic effectors that have been found in other oomycetes and that there is greater specificity in RXLR effectors than in CRN effectors.

### Time course for PhCRN effector expression during H. annuus * P. halstedii interactions

**Figure 5:**
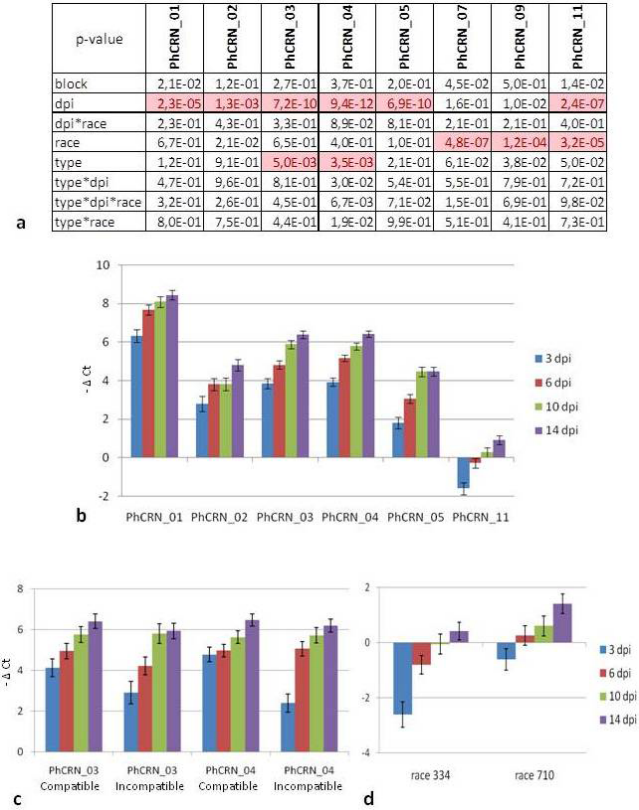
**Time course analysis by Q-RT-PCR of 8 putative PhCRN effectors**. a. Table of the p-values obtained by ANOVA analysis corresponding to following effects: block (for biological repetition), dpi (3, 6, 10 and 14), race (710 and 334), "type" (compatible: XRQ-334/PSC8-710 vs. incompatible: XRQ-710/PSC8-334), and the interactions between these 3 effects. Highlighted p-values are significant at Bonferonni test (α = 0.05 for the eight genes and for each tested effect). b. Time-course expression of 6 putative *P. halstedii *PhCRN effector genes, by Quantitative RT-PCR analysis, showing significant dpi effect. c. Time-course expression of 2 putative *P. halstedii *PhCRN effector genes, by Quantitative RT-PCR analysis, showing significant type effect. d. Time-course expression of putative *P. halstedii *PhCRN11 effector gene, by Quantitative RT-PCR analysis, showing significant race effect. (b, c and d) Mean expression values (- ΔCt) and standard errors are given from three independent biological experiments. In each biological experiment, the samples of three replicates were pooled before Quantitative RT-PCR assay.

To validate the *in planta *expression of some of these putative effectors, time course expression experiments were performed for 8 of them using quantitative RT-PCR (qRT-PCR). cDNA samples were obtained from three independent inoculation tests from two genotypes (XRQ and PSC8) infected with two *P. halstedii *races (710 and 334), which induced incompatible (XRQ/710 and PSC8/334) and compatible (XRQ/334 and PSC8/710) interactions. In the non-inoculated plant samples (control), no expression was detected with the set of primers that was used, suggesting *P. halstedii *specificity. In the inoculated plant samples, time course expression values (-ΔCt) were calculated and tested for statistical significance using ANOVA with the following variation factors: days post inoculation (dpi), type of interaction (compatible vs. incompatible), and *P. halstedii *race (Figure 5a).

The time course response was found highly statistically significant as stated by ANOVA (see Methods and Figure 5a), with a general increase in expression observed between 3 and 14 dpi for PhCRN_01, PhCRN_02, PhCRN_03, PhCRN_04, and PhCRN_05, whereas PhCRN_11 was repressed at 3 dpi and was subsequently expressed at the same level as the control genes (-ΔCt close to 0, Figure 5b). In contrast, the expression of PhCRN_07 and PhCRN_09 did not vary significantly according to inoculation time but did show a differential induction between 710 and 334 races (Figure 5a).

A difference in the time course of expression between compatible and incompatible interactions was found for PhCRN_03 and PhCRN_04, which were less expressed at 3 dpi (see Material and Method for statistical significance) in incompatible than in compatible interactions (Figures 5a and 5c). A statistically significant difference in the expression levels during infection between race 334 and race 710 was found for PhCRN_11 that was independent of the type of interaction, suggesting that this effector could play a role in the aggressiveness of the race (Figures 5a and 5d).

### Search for polymorphic sites in effector sequences for the four races of *P. halstedii*

Using the available sequences from the four *P. halstedii *races, specific assemblies were built for each race (see Methods) based on the highest frequency of alleles at each nucleic acid position. These results were used to search for polymorphisms in the PhCRN and PhRXLR DNA and predicted protein sequences (Table 1; nucleic acid and predicted peptide alignments are provided in Additional file 12).

**Table 1:** Polymorphism nucleic and amino acid site detection for 7 putative CRN observed on four *P.halstedii *races (100, 304, 703, and 710)

Cluster	Motif	Motif position	Aminoacid polymorphism	Aminoacid position	Nucleic acid polymorphism	SNP position
PhCRN_01	LRLFLAK	58	V/A	131	T/C	394
			Synonymous		T/C	440
			E/G	261	A/G	784
			I/F	264	A/T	792
			E/D	281	A/T	845
			M/V	441	A/G	1323

PhCRN_03	LELSLAK	56	R/K	373	A/G	1122

PhCRN_04	LELSLAK	58	Synonymous		C/T	1781
			Synonymous		A/G	1937
			T/L*	508	A/C	1953
					C/T	1954
			L/I*	519	C/A	1986
			Synonymous		A/G	2060
			A/D	558	C/A	2104
			L/P	568	T/C	2133
					T/C	2134

PhCRN_05	LQLFLAK	57	G/K	37	G/A	131
					G/A	132
			Synonymous		G/T	823
			Synonymous		A/T	946
			Synonymous		T/A	948

PhCRN_06	IELFLSK	59	Synonymous		T/C	1705
			Synonymous		G/A	1706
			Synonymous		C/T	1712
			Synonymous		G/A	1714
			Synonymous		G/A	1720
			I/T	505	T/C	1722
			E/K	506	G/A	1724
			L/T	508	C/A	1730
					T/C	1731
			L/I	519	C/A	1763
			Synonymous		C/T	1816
			Synonymous		A/G	1837
			G/C	555	G/T	1871
			A/E	558	C/A	1881
			A/T	561	G/A	1889
			Synonymous		A/G	1900
			I/F	578	A/T	1940
			A/V	579	C/T	1944
			Synonymous		A/G	1975

PhCRN_07	LKLSLAK	56	N/S	188	A/G	565
			L/W	258	T/G	775
			Synonymous		T/C	1025

PhCRN_09	LELSLAK	50	Synonymous		G/T	943
			Synonymous		A/G	1084

*: polymorphism within the race 710 only

Seven of the 15 putative CRNs were found to exhibit polymorphisms at the DNA level with a relatively high frequency of non-synonymous changes, leading to modification of the proteins' composition that may result in an alteration of the proteins' function or conformation. This could indicate a likely change in the effector profile of the race. As seen in Table 1, some clusters contained several polymorphic sites.

In contrast, none of the tested RXLR proteins were found to be polymorphic. This might be due to lower read counts in the different races for these potential effectors. Additionally, the search for polymorphisms was performed using effector transcript sequences, and it is possible that non-coding upstream sequences are more polymorphic. Alternatively, this could mean that the variability in CRN and RXLR does not expand in the same manner; CRN effectors appear to be more conserved across species but to present higher variability within species, whereas RXLR effectors are less conserved between species but show reduced variability within species.

### Inter-racial and Intra-racial polymorphisms

These polymorphisms were detected by comparing the predicted peptide sequences of the different races based on the most frequent allele detected for each race. However, this does not mean that each race has a unique profile. For example, non-synonymous polymorphisms for PhCRN_4 are observed within race 710 (Table 1). Intra-racial polymorphisms seemed to be the general pattern: as shown in Table 2, synonymous or non-synonymous SNPs are both (i) frequently observed within a race and (ii) associated with significant or non-significant differences between races, depending on the case.

**Table 2:** Inter and Intra-racial polymorphisms for 4 putative CRN observed on four *P.halstedii *races (100, 304, 703, and 710)

Cluster	Polymorphism site position	SNP allele	Race 100	Race 304	Race 703	Race 710	Khi-2
**PhCRN_01**	**1323**	A	3	6	27	8	20.88***

**PhCRN_03**	**1989**	G	6	15	6	16	98.81***
		T	1	17	0	19	
	**1989**	C	6	22	150	11	98.81***
	**2009**	A	6	25	100	25	3.49
	**2009**	T	1	4	12	0	3.49
	**2012**	T	7	16	100	5	24.91***
	**2012**	C	0	12	23	11	24.91***
	**649**	--	8	15	16	10	18.15***

**PhCRN_04**	**1122**	TA	3	6	2	21	2.21
		A	10	18	20	16	
	**1122**	G	13	25	16	24	2.21
	**2104**	A	2	7	2	12	7.82*

**PhCRN_05**	**2123**	C	8	12	13	10	9.22*
		A	3	12	2	13	
	**2123**	G	4	8	12	8	9.22*
	**2133**	TT	5	7	9	5	
	**2133**	CC	2	12	4	12	7.15
	**823**	T	11	24	0	0	56.00***
		G	0	0	11	10	
							

Frequencies for each allele for each race are indicated. The khi-2 calculations refer to H0: similar frequencies between races at each position (df = 3, *: significative at 5%; **: significative at 1%; ***: significative at 0.1%).

There are several possible explanations for this result. It may represent polymorphisms within a race that are due to one heterozygous genotype (where the allele frequencies are close to 50%), a mixture of homozygous genotypes or a mixture of these two situations, with the last possibility being more likely based on the frequencies that were observed. Alternatively, this result could be related to the definition of a "race", which is not a clonal genotype but an isolate showing particular differential responses when inoculated into a set of selected sunflower lines [2]. Whatever the case, intra-racial polymorphisms may not be linked to interactions between these races and the nine different sunflower lines that are usually used to differentiate *P. halstedii *races. However, including other genetic backgrounds on the host side could allow for better discrimination of pathogen intra-racial polymorphisms.

**Figure 6:**
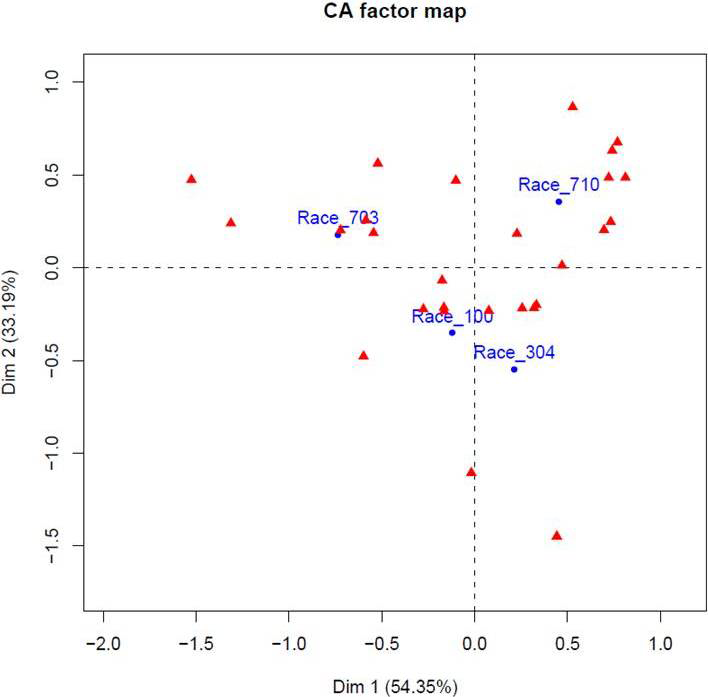
**Factorial correspondence analysis (FCA) performed on the 4 races based on the 22 SNPs observed within putative effector sequences**. Dudi.coa function (R software, ade4 package) was used to construct the CA factor map. The data table used for FCA was based on the absolute frequency observed for each CRN SNP (in red) for each race (100, 304, 703 and 710, in blue).

A factorial correspondence analysis (FCA) was performed on a data table (Additional file 13) in which each cell contains the number of reads obtained for each of the four *P. halstedii *races and each CRN SNP as a combination of the CRN effector, SNP positions leading to a non-synonymous variation, and amino acid substitutions (Figure 6). This analysis makes it possible to visualize the genetic distances between the four races based on their SNPs. The genetic distances between the three races 100, 703 and 710 and the FCA diagram indicate significant differences, in accordance with the hypothesis of three genetically differentiated groups of *P. halstedii *races [10]. Certain SNPs could allow for clear differentiation between race 304 and race 100 (Figure 6), which has not been observed previously [10].

The use of these new SNP markers located in putative effector sequences should provide an additional tool to extend the polymorphism analyses allowed by the 12 previously published EST-derived markers [11]. They should also lead to a better definition of what the genetic structure of a "race" is and allow for better discrimination between races, which will make it possible to re-evaluate the genetic structure and evolution of populations of *P. halstedii*. Moreover, SNP markers are considered the most useful markers in diploid organisms because they are co-dominant, specific and easy to use with new genotyping techniques. It is important to extend this work in the future by sequencing additional races and identifying more SNPs in putative effector sequences.

## Conclusions

This study represents a substantial improvement of existing knowledge regarding *P. halstedii *sequences that are expressed during the interaction of this species with sunflowers. This work also reveals infection mechanisms similar to those observed in other oomycetes and the presence of putative RXLR and CRN effectors. Using polymorphic sites in CRN effector sequences, the observed genetic distances between three races (100, 703 and 710) were shown to be in agreement with the conclusions of Delmotte et al. [10]. Certain SNPs might allow for clear differentiation between races 304 and 100 (Figure 5), which has not been detected previously [10].

To improve the knowledge regarding the genetic structure of *P. halstedii *populations, it is necessary to obtain polymorphism data for all of the recorded races and to include different geographical isolates for each race, particularly because intra-racial polymorphisms appear to be significant in this study. These results increase interest in reassessing the current classification of *P. halstedii *isolates based on a description other than their interaction with different sunflower lines. This re-evaluation could be performed using a larger set of molecular markers, particularly SNPs, that occur mainly in effector sequences, which should be under higher selective pressure.

## Methods

### Plant and oomycete materials

Two inbred sunflower lines, XRQ (resistant line) and PSC8 (susceptible line), were infected with *Plasmopara halstedii *(race 710) in a confined culture chamber using an infection method previously described by Mouzeyar et al. [40]. Entire plants were harvested at 14 dpi and were immediately frozen in liquid nitrogen and stored at -80°C.

*P. halstedii *spores (from races 100, 304, 703 and 710) were provided by Dr. D. Tourvieille de Labrouhe (INRA Clermont-Ferrand, FRANCE). They were collected from the inbred sunflower line GB (susceptible to all races) in a confined culture chamber with the same infection method described above [40]. Cotyledons covered with dense whitish fluffy growth of zoosporangia and sporangiophores were placed in water and shaken to separate oomycete material from the sunflower cotyledons. The liquid containing the sporangia was centrifuged at 2,000 × g, and the supernatant was removed to concentrate the solution.

qRT-PCR analyses were performed using the BioMark™ system (Fluidigm corporation, CA, USA). The XRQ and PSC8 plants were inoculated with *P. halstedii *race 710 as previously described, leading to either an incompatible or compatible interaction, respectively. They were also independently infected with race 334 (XRQ susceptible, PSC8 resistant) with three replicates. The aerial portions of the plants were sampled at 3, 6, 10 and 14 dpi and immediately frozen in liquid nitrogen.

### DNA extraction

DNA was extracted using the DNeasy mini kit (Qiagen USA, Valencia, CA). DNA quantity and quality was estimated by TAE gel electrophoresis.

### RNA extraction

RNA was extracted with the Qiagen RNeasy Midi Kit (Qiagen USA, Valencia, CA), and the quantity of RNA was estimated using an ND-1000 Spectrophotometer (NanoDrop USA, Wilmington, DE). RNA quality was verified using an Agilent Bioanalyzer 2100 LabChip and an Agilent RNA 6000 Nano kit (Agilent Technologies, USA) as well as being evaluated on a 2% agarose gel stained with ethidium bromide.

### cDNA synthesis and normalization

For this step, the protocol described by Novaes et al. [24] was followed with minor modifications. cDNA was synthesized from 1-2 μg of RNA, and the incorporated adaptors were removed *in silico *after sequencing.

### 454 sequencing and assembly

Two samples, infected XRQ and infected PSC8 (each containing approximately 14 μg of normalized cDNA), were sent to the EPGV team at Evry (France) for library construction and sequencing at CNS Evry (France).

The sequencing runs were performed on a Roche 454 GS-FLX TITANIUM sequencer following the manufacturer's recommendations. An initial filtering was performed on base quality: reads shorter than 50 nucleotides were removed, and of the remaining reads, those that contained more than 50% Ns were also removed. The sequences were cleaned up to remove adaptor sequences *in silico*. All of the reads obtained are available at ENA (European Nucleotide Archive [41]), accession #s ERP000522, ERS023538, ERS023539, ERX010280, ERX010281 and from ERR029545 to ERR029553). Clustering was carried out with a modified version of TGICL [42] permitting parallelization on SGE computer clusters and performing data pre-processing to remove redundancy (using the nrcl and tclust software provided in the TGICL package). TGICL (-p 97 -l 40) was run on the cleaned data generated in this study merged with *H. annuus *sequences available from public domains (January 2009). For polymorphism analyses, longer PhCRN and PhRXLR sequences were obtained from a new clustering assembly performed on HP and *H. annuus *sequences supplemented with 800,000 new, cleaned cDNA sequences. These supplementary sequences were obtained from independent 454 sequencing of cDNAs from the susceptible sunflower line GB inoculated with the 4 races and harvested at 10 to 14 dpi using the same clustering protocol as before. Approximately 211,000 clusters and singletons were generated (with an average length of 447 bp and a median length of 378 bp) (unpublished data from Delmotte and col.)

### Heliagene database

Heliagene (http://www.heliagene.org) is a bioinformatic portal that was developed to analyze *Helianthus sp*. EST data found in public databases (January 2008). Heliagene provides a variety of pre-computed analyses and tools for EST clusters and for exploring gene function and protein families in a user-friendly fashion. The HP portal was created on the same principle (using *Helianthus sp*. EST data found in public databases) but also includes publically available sequences of *P. halstedii *and 454 reads provided by this work.

### Creation of oomycete (OOM) databases and searches for RXLR and CRN motifs

A database containing 345,155 sequences from oomycetes represented by Phytophthora (220,253), Pythium (105,043), Hyaloperonospora (14,589), Aphanomyces (3602), Saprognelia (1513) and Plasmopara (155) has been built to classify the HP expressed sequences. In addition to the mRNA sequences available from NCBI, this database includes gene models predicted from the genomic sequences of *Hyaloperonospora arabidopsidis *that were made available to the scientific community in December 2010 [30]. The OOM database is available for BLAST queries at http://www.heliagene.org/HP.

The search for matches with the RXLR and CRN effectors was performed with PSI-TBLASTN (PSSM) using the annotated sequences that were available in NCBI in March 2010 as models for each type of effector [35].

### Primer design and PCR amplification

Primer pairs were designed using Primer3 (Tm = 60°C ± 1, 20 nucleotide primer length, 100-400 amplicon length) [43]. PCR amplifications were performed with 20 ng of DNA, 75 μM each dNTP, 0.75 U of Taq DNA polymerase (GoTaq, Promega), 1X* Taq Polymerase buffer and 0.6 μM each primer. Amplification was carried out in a Mastercycler pro S Eppendorf thermocycler using 46 cycles of 94°C for 30 s, 60°C for 30 s and 72°C for 50 s. The obtained PCR products were separated by TAE agarose gel electrophoresis.

### Polymorphism detection

First, a sequence assembly was obtained independently for each *P. halstedii *race using CAP3 [44] with overlap percent identity cutoff parameter set to 90% and other parameters let to default values. Then, the predicted peptide from the cluster was used as a query in TBLASTN searches with these assemblies as targets. The predicted peptide sequences for the races were then aligned together with the predicted peptide sequence of the cluster to detect non-synonymous variations. Only the polymorphisms that were found within the region that exhibited the highest level of similarity between the races were selected, to account for the fact that other variations could be due to errors in 454 sequencing. Finally, the nucleotide sequence of each race was used as a query in BLASTX against the peptide prediction of the cluster as a target to identify SNP positions.

### Time course analysis of putative PhCRN effectors by qRT-PCR

Total RNA from aerial portions of inoculated XRQ and PSC8 plants were extracted, and first strand cDNAs were synthesized from 1 μg of total RNA using Transcriptor Reverse Transcriptase (Roche Applied Science, Indianapolis, IN, USA) and oligonucleotide d17T-V primers following the manufacturer's recommendations. qRT-PCR was performed using Fluidigm™ technology following Spurgeon et al., [45] in a 96*96-well plate. Primers were designed using Primer3 (Tm = 60°C ± 1, 20 nucleotide primer length, 120-200 amplicon length). The results were subjected to quality assessment, and the obtained fluorescence data were converted to Cycle threshold (Ct) values using Fluidigm Real-Time PCR Analysis Software version 3. Using two reference *P. halstedii *genes (AY773346.1, an internal transcribed spacer, and a gene encoding a ribosomal protein, L13e), a ΔCt value for each sample was calculated by subtracting the mean Ct value of the reference genes from the Ct value of each gene. ANOVA was then performed to test for significance (see Methods - statistical analysis below).

### Statistical analysis

FCA was performed using R (version 2.9.2, function dudi.coa from the ade4 package). For the quantitative RT-PCR time course experiments, ANOVA was performed employing the SAS GLM procedure for each gene with a test for each potential effect (race of *P. halstedii*, type of interaction, days post inoculation) and their interactions. Statistical significance was checked based on a Family-Wise Error Rate of 5% for each effect, leading to a p-value cut-off of 0.00625 for each gene*effect combination.

## Authors' contributions

FA synthesized and normalized the cDNA, analyzed the data, identified the SNPs and wrote the manuscript. SC, TH & JG assembled the 454 sequences, annotated the sequences, ran PSI-BLAST, and created the database. MCLP & DB generated and summarized the sequencing results from the 454 sequencing platform. AB assisted in the analysis of the effectors and the plant-specific sequences. QG, DR and MCB generated samples for qRT-PCR, performed the Fluidigm experiment and analyzed the data. LG assisted in analysis of the data and in manuscript preparation. PV was responsible for coordination of the study, analysis of the EST data and manuscript preparation. All authors read and approved the final manuscript.

## Supplementary Material

Additional file 1 **List of 405 specific oomycete clusters**. Contains cluster length, numbers of reads in XRQ and PSC8 samples, tested by PCR amplification and best hit on different databases (InterPro, Go, TAIR and OOM). Click here for file

Additional file 2 **405 specific oomycete clusters sequences**. In fasta format. Click here for file

Additional file 3 **List of 172 non specific oomycete clusters**. Contains cluster length, numbers of reads in XRQ and PSC8 samples, tested by PCR amplification and best hit on different databases (InterPro, Go, TAIR and OOM). Click here for file

Additional file 4 **172 non specific oomycete clusters sequences**. In fasta format. Click here for file

Additional file 5 **List of 52 putative oomycete verified by PCR amplification**. Contains PCR amplification results on *H. annuus *and *P. halstedii *DNA, PCR product size, primer sequences used and origin of the selected HP (Oomycete specific or oomycete non specific). Click here for file

Additional file 6 **List of 130 specific plant clusters (with "defense", "disease" and "pathogenesis" keyword search)**. Contains cluster length, PCR amplification results on *H. annuus *and *P. halstedii *DNA, PCR product size, primer sequences used and best hit results on InterPro, Go and TAIR database. Click here for file

Additional file 7 **22 specific *Helianthus annuus *clusters sequences identified by PCR**. In fasta format. Click here for file

Additional file 8 **List of 20 putative RXLR and CRN effectors found by PSI-BLAST (E-value 1e-04)**. Contains cluster length, number of reads, motif found, signal peptide presence probability, its sequence and InterPro Scan results. Click here for file

Additional file 9 **20 putative RXLR and CRN effector sequences found by PSI-BLAST**. In fasta format. Click here for file

Additional file 10 **20 putative RXLR and CRN effectors predicted peptide sequences**. In fasta format. Click here for file

Additional file 11 **15 putative CRN effectors best hits against OOM (with E-value and % Identity)**. Click here for file

Additional file 12 **Alignments of nucleic acid and predicted peptides of PhCRN putative effectors showing polymorphisms**. Click here for file

Additional file 13 **FCA analysis dataset for the polymorphic *P. halstedii *putative effectors**. Contains the original dataset (number of reads presenting each SNP for each race) and FCA analysis results (row coordinates, weight coordinates and column coordinates). Click here for file

## References

[B1] Virányi F The Sunflower--Plasmopara Halsted II Pathosystem: Natural and Artificially Induced Coevolution Advances in Downy Mildew Research 2002 167 172

[B2] Tourvieille de Labrouhe D Pilorgé E Nicolas P Vear F Le mildiou du tournesol Paris: CETIOM, INRA 2000

[B3] Vear F Gentzbittel L Philippon J Mouzeyar S Mestries E Roeckel-Drevet P Tourvieille de Labrouhe D Nicolas P The genetics of resistance to five races of downy mildew (Plasmopara halstedii) in sunflower (Helianthus annuus L.) Theoretical and Applied Genetics 1997 95 584 589 10.1007/s001220050599

[B4] Radwan O Bouzidi MF Vear F Identification of non-TIR-NBS-LRR markers linked to the Pl5/Pl8 locus for resistance to downy mildew in sunflower Theoretical and applied genetics 2003 106 1438 46 12750787 10.1007/s00122-003-1196-1

[B5] Radwan O Mouzeyar S Nicolas P Bouzidi MF Induction of a sunflower CC-NBS-LRR resistance gene analogue during incompatible interaction with Plasmopara halstedii Journal of experimental botany 2005 56 567 75 10.1093/jxb/eri030 15545294

[B6] Radwan O Gandhi S Heesacker A Whitaker B Taylor C Plocik A Kesseli R Kozik A Michelmore RW Knapp SJ Genetic diversity and genomic distribution of homologs encoding NBS-LRR disease resistance proteins in sunflower Molecular genetics and genomics 2008 280 111 25 10.1007/s00438-008-0346-1 18553106

[B7] Vear F Serre F Jouan-Dufournel I Bert PF Roche S Walser P Tourvieille de Labrouhe D Vincourt P Inheritance of quantitative resistance to downy mildew (Plasmopara halstedii) in sunflower (Helianthus annuus L.) Euphytica 2008 164 561 570 10.1007/s10681-008-9759-5

[B8] Bouzidi MF Parlange F Nicolas P Mouzeyar S Expressed Sequence Tags from the oomycete Plasmopara halstedii, an obligate parasite of the sunflower BMC microbiology 2007 7 110 10.1186/1471-2180-7-110 18062809 PMC2242796

[B9] Roeckel-Drevet P Tourvieille J Drevet JR Says-Lesage V Nicolas P Tourvieille de Labrouhe D Development of a polymerase chain reaction diagnostic test for the detection of the biotrophic pathogen Plasmopara halstedii in sunflower Canadian journal of microbiology 1999 45 797 803 10526404 10.1139/w99-068

[B10] Delmotte F Giresse X Richard-Cervera S M'Baya J Vear F Tourvieille J Walser P Tourvieille de Labrouhe D Single nucleotide polymorphisms reveal multiple introductions into France of Plasmopara halstedii, the plant pathogen causing sunflower downy mildew Infection, genetics and evolution 2008 8 534 40 10.1016/j.meegid.2008.02.012 18450523

[B11] Giresse X Tourvieille de Labrouhe D Richard-Cervera S Delmotte F Twelve polymorphic expressed sequence tags-derived markers for Plasmopara halstedii, the causal agent of sunflower downy mildew Molecular Ecology Notes 2007 7 1363 1365 10.1111/j.1471-8286.2007.01887.x

[B12] Hardham AR Cell biology of plant-oomycete interactions Cellular microbiology 2007 9 31 9 10.1111/j.1462-5822.2006.00833.x 17081190

[B13] Lamour KH Win J Kamoun S Oomycete genomics: new insights and future directions FEMS microbiology letters 2007 274 1 8 10.1111/j.1574-6968.2007.00786.x 17559387

[B14] Coates ME Beynon JL Hyaloperonospora arabidopsidis as a pathogen model Annual review of phytopathology 2010 48 329 45 10.1146/annurev-phyto-080508-094422 19400636

[B15] Lebeda A Sedlářová M Petřivalský M Prokopová J Diversity of defence mechanisms in plant-oomycete interactions: a case study of Lactuca spp. and Bremia lactucae European Journal of Plant Pathology 2008 122 71 89 10.1007/s10658-008-9292-3

[B16] Kamoun S Groovy times: filamentous pathogen effectors revealed Current opinion in plant biology 2007 10 358 65 10.1016/j.pbi.2007.04.017 17611143

[B17] Hogenhout SA Van der Hoorn RAL Terauchi R Kamoun S Emerging concepts in effector biology of plant-associated organisms Molecular plant-microbe interactions 2009 22 115 22 10.1094/MPMI-22-2-0115 19132864

[B18] Oliva R Win J Raffaele S Boutemy L Bozkurt TO Chaparro-Garcia A Segretin ME Stam R Schornack S Cano LM van Damme M Huitema E Thines M Banfield MJ Kamoun S Recent developments in effector biology of filamentous plant pathogens Cellular microbiology 2010 12 705 15 10.1111/j.1462-5822.2010.01471.x 20374248

[B19] Kamoun S A catalogue of the effector secretome of plant pathogenic oomycetes Annual review of phytopathology 2006 44 41 60 10.1146/annurev.phyto.44.070505.143436 16448329

[B20] Birch PRJ Rehmany AP Pritchard L Kamoun S Beynon JL Trafficking arms: oomycete effectors enter host plant cells Trends in microbiology 2006 14 4 8 10.1016/j.tim.2005.11.004 16356717

[B21] Rehmany AP Gordon A Rose LE Allen RL Armstrong MR Whisson SC Kamoun S Tyler BM Birch PRJ Beynon JL Differential Recognition of Highly Divergent Downy Mildew Avirulence Gene Alleles by RPP1 Resistance Genes from Two Arabidopsis Lines The Plant Cell 2005 17 1839 1850 10.1105/tpc.105.031807 15894715 PMC1143081

[B22] Tyler BM Entering and breaking: virulence effector proteins of oomycete plant pathogens Cellular microbiology 2009 11 13 20 10.1111/j.1462-5822.2008.01240.x 18783481

[B23] Torto TA Li S Styer A Huitema E Testa A Gow NAR van West P Kamoun S EST mining and functional expression assays identify extracellular effector proteins from the plant pathogen Phytophthora Genome research 2003 13 1675 85 10.1101/gr.910003 12840044 PMC403741

[B24] Novaes E Drost DR Farmerie WG Pappas JG Grattapaglia D Sederoff RR Kirst M High-throughput gene and SNP discovery in Eucalyptus grandis, an uncharacterized genome BMC genomics 2008 9 312 10.1186/1471-2164-9-312 18590545 PMC2483731

[B25] Cheung F Win J Lang JM Hamilton J Vuong H Leach JE Kamoun S Lévesque CA Tisserat N Buell CR Analysis of the Pythium ultimum transcriptome using Sanger and Pyrosequencing approaches BMC genomics 2008 9 542 10.1186/1471-2164-9-542 19014603 PMC2612028

[B26] Schafleitner R Tincopa LR Palomino O Rossel G Robles RF Alagon R Rivera C Quispe C Rojas L Pacheco JA Solis J Cerna D Kim JY Hou J Simon R A sweetpotato gene index established by de novo assembly of pyrosequencing and Sanger sequences and mining for gene-based microsatellite markers BMC genomics 2010 11 604 10.1186/1471-2164-11-604 20977749 PMC3017860

[B27] Margulies M Egholm M Altman WE Attiya S Bader JS Bemben LA Berka J Braverman MS Chen Y-J Chen Z Dewell SB Du L Fierro JM Gomes XV Godwin BC He W Helgesen S He Ho C Irzyk GP Jando SC Alenquer MLI Jarvie TP Jirage KB Kim J-B Knight JR Lanza JR Leamon JH Lefkowitz SM Lei M Li J Lohman KL Lu H Makhijani VB McDade KE McKenna MP Myers EW Nickerson E Nobile JR Plant R Puc BP Ronan MT Roth GT Sarkis GJ Simons JF Simpson JW Srinivasan M Tartaro KR Tomasz A Vogt KA Volkmer GA Wang SH Wang Y Weiner MP Yu P Begley RF Rothberg JM Genome sequencing in microfabricated high-density picolitre reactors Nature 2005 437 376 80 16056220 10.1038/nature03959 PMC1464427

[B28] Mardis ER The impact of next-generation sequencing technology on genetics Trends in genetics 2008 24 133 41 10.1016/j.tig.2007.12.007 18262675 PMC2680276

[B29] PlantGDB - Resources for Plant Comparative Genomics http://www.plantgdb.org/

[B30] Baxter L Tripathy S Ishaque N Boot N Cabral A Kemen E Thines M Ah-Fong A Anderson R Badejoko W Bittner-Eddy P Boore JL Chibucos MC Coates M Dehal P Delehaunty K Dong SM Downton P Dumas B Fabro G Fronick C Fuerstenberg SI Fulton L Gaulin E Govers F Hughes L Humphray S Jiang RHY Judelson H Kamoun S Kyung K Meijer H Minx P Morris P Nelson J Phuntumart V Qutob D Rehmany A Rougon-Cardoso A Ryden P Torto-Alalibo T Studholme D Wang YC Win J Wood J Clifton SW Rogers J Van den Ackerveken G Jones JDG McDowell JM Beynon J Tyler BM Signatures of Adaptation to Obligate Biotrophy in the Hyaloperonospora arabidopsidis Genome Science 2010 330 1549 1551 10.1126/science.1195203 21148394 PMC3971456

[B31] Sierra R Rodríguez-R LM Chaves D Pinzon A Grajales A Rojas A Mutis G Cardenas M Burbano D Jimenez P Bernal A Restrepo S Discovery of Phytophthora infestans genes expressed in planta through mining of cDNA libraries PloS one 2010 5 e9847 10.1371/journal.pone.0009847 20352100 PMC2844423

[B32] Thines M Kamoun S Oomycete-plant coevolution: recent advances and future prospects Current opinion in plant biology 2010 13 427 33 10.1016/j.pbi.2010.04.001 20447858

[B33] Schornack S van Damme M Bozkurt TO Cano LM Smoker M Thines M Gaulin E Kamoun S Huitema E Ancient class of translocated oomycete effectors targets the host nucleus Proc Nat Acad Sci USA 2010 107 17421 17426 10.1073/pnas.1008491107 20847293 PMC2951462

[B34] Whisson SC Boevink PC Moleleki L Avrova AO Morales JG Gilroy EM Armstrong MR Grouffaud S van West P Chapman S Hein I Toth IK Pritchard L Birch PRJ A translocation signal for delivery of oomycete effector proteins into host plant cells Nature 2007 450 115 8 10.1038/nature06203 17914356

[B35] Altschul SF Madden TL Schäffer AA Zhang J Zhang Z Miller W Lipman DJ Gapped BLAST and PSI-BLAST: a new generation of protein database search programs Nucleic acids research 1997 25 3389 402 10.1093/nar/25.17.3389 9254694 PMC146917

[B36] Lévesque CA Brouwer H Cano L Hamilton JP Holt C Huitema E Raffaele S Robideau GP Thines M Win J Zerillo MM Beakes GW Boore JL Busam D Dumas B Ferriera BOLD Fuerstenberg S Gachon CMM Gaulin E Govers F Grenville-Briggs L Horner N Hostetler J Jiang RHY Johnson J Haining Lin TK Meijer HJG Moore B Morris P Phuntmart V Puiu D Shetty J Stajich JE Tripathy S Wawra S van West P Whitty BR Coutinho PM Martin BHF Thomas PD Tyler BM De Vries RP Kamoun S Yandell M Tisserat N Buell CR Genome sequence of the necrotrophic plant pathogen Pythium ultimum reveals original pathogenicity mechanisms and effector repertoire Genome biology 2010 11 R73 10.1186/gb-2010-11-7-r73 20626842 PMC2926784

[B37] Corpet F Multiple sequence alignment with hierarchical clustering Nucleic acids research 1988 16 10881 90 10.1093/nar/16.22.10881 2849754 PMC338945

[B38] Haas BJ Kamoun S Zody MC Jiang RHY Handsaker RE Cano LM Grabherr M Kodira CD Raffaele S Torto-Alalibo T Bozkurt TO Ah-Fong AMV Alvarado L Anderson VL Armstrong MR Avrova A Baxter L Beynon J Boevink PC Bollmann SR Bos JIB Bulone V Cai GH Cakir C Carrington JC Chawner M Conti L Costanzo S Ewan R Fahlgren N Fischbach MA Fugelstad J Gilroy EM Gnerre S Green PJ Grenville-Briggs LJ Griffith J Grunwald NJ Horn K Horner NR Hu CH Huitema E Jeong DH Jones AME Jones JDG Jones RW Karlsson EK Kunjeti SG Lamour K Liu ZY Ma LJ MacLean D Chibucos MC McDonald H McWalters J Meijer HJG Morgan W Morris PF Munro CA O'Neill K Ospina-Giraldo M Pinzon A Pritchard L Ramsahoye B Ren QH Restrepo S Roy S Sadanandom A Savidor A Schornack S Schwartz DC Schumann UD Schwessinger B Seyer L Sharpe T Silvar C Song J Studholme DJ Sykes S Thines M van de Vondervoort PJI Phuntumart V Wawra S Weide R Win J Young C Zhou SG Fry W Meyers BC van West P Ristaino J Govers F Birch PRJ Whisson SC Judelson HS Nusbaum C Genome sequence and analysis of the Irish potato famine pathogen Phytophthora infestans Nature 2009 461 393 8 10.1038/nature08358 19741609

[B39] Trower MK Clark KG PCR cloning of a streptomycin phosphotransferase (aphE) gene from Streptomyces griseus ATCC 12475 Nucleic acids research 1990 18 4615 2167474 10.1093/nar/18.15.4615 PMC331307

[B40] Mouzeyar S Labrouhe DT Vear F Histopathological Studies of Resistance of Sunflower (Helianthus annuus L.) to Downy Mildew (Plasmopara halstedii) Journal of Phytopathology 1993 139 289 297 10.1111/j.1439-0434.1993.tb01430.x

[B41] European Nucleotide Archive | EBI http://www.ebi.ac.uk/ena/

[B42] Pertea G Huang X Liang F Antonescu V Sultana R Karamycheva S Lee Y White J Cheung F Parvizi B Tsai J Quackenbush J TIGR Gene Indices clustering tools (TGICL): a software system for fast clustering of large EST datasets Bioinformatics 2003 19 651 2 10.1093/bioinformatics/btg034 12651724

[B43] Rozen S Skaletsky H Primer3 on the WWW for General Users and for Biologist Programmers Bioinformatics Methods and Protocols: Methods in Molecular Biology 2000 365 386 10.1385/1-59259-192-2:365 10547847

[B44] Huang X Madan A CAP3: A DNA sequence assembly program Genome Res 1999 9 868 877 10.1101/gr.9.9.868 10508846 PMC310812

[B45] Spurgeon SL Jones RC Ramakrishnan R High throughput gene expression measurement with real time PCR in a microfluidic dynamic array PloS one 2008 3 e1662 10.1371/journal.pone.0001662 18301740 PMC2244704

[B46] Gouzy J Carrere S Schiex T FrameDP: sensitive peptide detection on noisy matured sequences Bioinformatics 2009 25 670 1 10.1093/bioinformatics/btp024 19153134 PMC2647831

