# Supplementary material for: Transcriptomic analysis of the interaction between Helianthus annuus and its obligate parasite Plasmopara halstedii shows single nucleotide polymorphisms in CRN sequences
Source: BMC Genomics. 2011 Oct 11;12:498. doi: 10.1186/1471-2164-12-498 (PMC3204308; doi:10.1186/1471-2164-12-498)
Supplement: Additional file 12 — Alignments of nucleic acid and predicted peptides of PhCRN putative effectors showing polymorphisms. [file 1471-2164-12-498-S12.PDF]

Additional file 12: Alignments of nucleic acid and predicted peptides of PhCRN putative effectors showing polymorphisms:

PhCRN	Nucleic acid alignment	Predicted peptide alignment
PhCRN_01	PhCRN_01_NA	PhCRN_01_AA
PhCRN_03	PhCRN_03_NA	PhCRN_03_AA
PhCRN_04	PhCRN_04_NA	PhCRN_04_AA
PhCRN_05	PhCRN_05_NA	PhCRN_05_AA
PhCRN_06	PhCRN_06_NA	PhCRN_06_AA
PhCRN_07	PhCRN_07_NA	PhCRN_07_AA
PhCRN_09	PhCRN_09_NA	PhCRN_09_AA

PhCRN_01_NA

```
                 261    270       280       290       300       310       320       330       340       350       360       370       380       390
                 |--------+---------+---------+---------+---------+---------+---------+---------+---------+---------+---------+---------+---------|
PhCRN_01_cluster GAGTTGGACGCTGTGGGTGCGCCACTTAATGTGGTTGGTTTATCGGAGACAGACGTGCAGTTTTTAGTTACAAAGGAACTTGCTAAGGCTAAAAAGACCCGTGTACACGTGCTGGTGGTGGTTCCGGAGG
   PhCRN_01-r304 GAGTTGGACGCTGTGGGTGCGCCACTTAATGTGGTTGGTTTATCGGAGACAGACGTGCAGTTTTTAGTTACAAAGGAACTTGCTAAGGCTAAAAAGACCCGTGTACACGTGCTGGTGGTGGTTCCGGAGG
   PhCRN_01-r710 GAGTTGGACGCTGTGGGTGCGCCACTTAATGTGGTTGGTTTATCGGAGACAGACGTGCAGTTTTTAGTTACAAAGGAACTTGCTAAGGCTAAAAAGACCCGTGTACACGTGCTGGTGGTGGTTCCGGAGG
   PhCRN_01-r100                               GTGGTTGGTTTATCGGAGACAGACGTGCAGTTTTTAGTTACAAAGGAACTTGCTAAGGCTAAAAAGACCCGTGTACACGTGCTGGTGGTGGTTCCGGAGG
   PhCRN_01-r703                                                                                                                               GG
       Consensus gagttggacgctgtgggtgcgccacttaatgtggttggtttatcggagacagacgtgcagtttttagttacaaaggaacttgctaaggctaaaaagacccgtgtacacgtgctggtggtggttccggaGG

                 391    400       410       420       430       440       450       460       470       480       490       500       510       520
                 |--------+---------+---------+---------+---------+---------+---------+---------+---------+---------+---------+---------+---------|
PhCRN_01_cluster GGGTCAGCAGAACGGAAGTGTGTGACATGATACGGGACGGACGAGGCATTCGGAAGTTCAACGAACCACTTGGGGATTCCGAGTTGGAATATGAAGGTGGAGTTCCTGCAACATTCAAGACGTTGCGAAA
   PhCRN_01-r304 GGGTCAGCAGAACGGAAGTGTGTGACATGATACGGGACGGACGAGGCATTCGGAAGTTCAACGAACCACTTGGGGATTCCGAGTTGGAATATGAAGGTGGAGTTCCTGCAACATTCAAGACGTTGCGAAA
   PhCRN_01-r710 GGGTCAGCAGAACGGAAGTGTGTGACATGATACGGGACGGACGAGGCATTCGGAAGTTCAACGAACCACTTGGGGATTCCGAGTTGGAATATGAAGGTGGAGTTCCTGCAACATTCAAGACGTTGCGAAA
   PhCRN_01-r100 GGGCCAGCAGAACGGAAGTGTGTGACATGATACGGGACGGACGAGGCATCCGGAAGTTCAACGAACCACTTGGGGATTCCGAGTTGGAATATGAAGGTGGAGTTCCTGCAACATTCAAGACGTTGCGAAA
   PhCRN_01-r703 GGGTCAGCAGAACGGAAGTGTGTGACATGATACGGGACGGACGAGGCATTCGGAAGTTCAACGAACCACTTGGGGATTCCGAGTTGGAATATGAAGGTGGAGTTCCTGCAACATTCAAGACGTTGCGAAA
       Consensus GGGtCAGCAGAACGGAAGTGTGTGACATGATACGGGACGGACGAGGCATtCGGAAGTTCAACGAACCACTTGGGGATTCCGAGTTGGAATATGAAGGTGGAGTTCCTGCAACATTCAAGACGTTGCGAAA

                 521    530       540       550       560       570       580       590       600       610       620       630       640       650
                 |--------+---------+---------+---------+---------+---------+---------+---------+---------+---------+---------+---------+---------|
PhCRN_01_cluster AGCCACTGTGGCCGCATTTGCAAGTCTTCTCGATCGCATACCAGTGATATTCATCCGCGCTCCCCCTTTGTCCGGAAAGACGGCCTTGTGCGATCTACTCTACGACCACATTGTTCGCTCCAAGCCTGAT
   PhCRN_01-r304 AGCCACTGTGGCCGCATTTGCAAGTCTTCTCGATCGCATACCAGTGATATTCATCCGCGCTCCCCCTTTGTCCGGAAAGACGGCCTTGTGCGATCTACTCTACGACCACATTGTTCGCTCCAAGCCTGAT
   PhCRN_01-r710 AGCCACTGTGGCCGCATTTGCAAGTCTTCTCGATCGCATACCAGTGATATTCATCCGCGCTCCCCCTTTGTCCGGAAAGACGGCCTTGTGCGATCTACTCTACGACCACATTGTTCGCTCCAAGCCTGAT
   PhCRN_01-r100 AGCCACTGTGGCCGCATTTGCAAGTCTTCTCGATCGCATACCAGTGATATTCATCCGCGCTCCCCCTTTGTCCGGAAAGACGGCCTTGTGCGATCTACTCTACGACCACATTGTTCGCTCCAAGCCTGAT
   PhCRN_01-r703 AGCCACTGTGGCCGCATTTGCAAGTCTTCTCGATCGCATACCAGTGATATTCATCCGCGCTCCCCCTTTGTCCGGAAAGACGGCCTTGTGCGATCTACTCTACGACCACATTGTTCGCTCCAAGCCTGAT
       Consensus AGCCACTGTGGCCGCATTTGCAAGTCTTCTCGATCGCATACCAGTGATATTCATCCGCGCTCCCCCTTTGTCCGGAAAGACGGCCTTGTGCGATCTACTCTACGACCACATTGTTCGCTCCAAGCCTGAT

                 651    660       670       680       690       700       710       720       730       740       750       760       770       780
                 |--------+---------+---------+---------+---------+---------+---------+---------+---------+---------+---------+---------+---------|
PhCRN_01_cluster GCACTTGTTTCCCGCATTCTGGCCAATCAAATGCCTGTAGATGGCAAGTTTACCGAGTTTTTCAAATTTTTGTACGGCTATGAGTTTGAAGCATTCTGCGCACACAAAGGCGATCGGGTGGTACTGATTG
   PhCRN_01-r304 GCACTTGTTTCCCGCATTCTGGCCAATCAAATGCCTGTAGATGGCAAGTTTACCGAGTTTTTCAAATTTTTGTACGGCTATGAGTTTGAAGCATTCTGCGCACACAAAGGCGATCGGGTGGTACTGATTG
   PhCRN_01-r710 GCACTTGTTTCCCGCATTCTGGCCAATCAAATGCCTGTAGATGGCAAGTTTACCGAGTTTTTCAAATTTTTGTACGGCTATGAGTTTGAAGCATTCTGCGCACACAAAGGCGATCGGGTGGTACTGATTG
   PhCRN_01-r100 GCACTTGTTTCCCGCATTCTGGCCAATCAAATGCCTGTAGATGGCAAGTTTACCGAGTTTTTCAAATTTTTGTACGGCTATGAGTTTGAAGCATTCTGCGCACACAAAGGCGATCGGGTGGTACTGATTG
   PhCRN_01-r703 GCACTTGTTTCCCGCATTCTGGCCAATCAAATGCCTGTAGATGGCAAGTTTACCGAGTTTTTCAAATTTTTGTACGGCTATGAGTTTGAAGCATTCTGCGCACACAAAGGCGATCGGGTGGTACTGATTG
       Consensus GCACTTGTTTCCCGCATTCTGGCCAATCAAATGCCTGTAGATGGCAAGTTTACCGAGTTTTTCAAATTTTTGTACGGCTATGAGTTTGAAGCATTCTGCGCACACAAAGGCGATCGGGTGGTACTGATTG

                 781    790       800       810       820       830       840       850       860       870       880       890       900       910
                 |--------+---------+---------+---------+---------+---------+---------+---------+---------+---------+---------+---------+---------|
PhCRN_01_cluster ATGAAGCTCAAATTACGTATAACGACGAGCAGCTGTGGAGGGGATACGTGAAAGGCGCATTGGAATCGCAAATACGAGGCCTTCGCTTCGTTCTCTTTTCATCGTATGGCAGCTTTAATATTTACCGCAA
   PhCRN_01-r304 ATGGAGCTCAATTTACGTATAACGACGAGCAGCTGTGGAGGGGATACGTGAAAGGCGCATTGGATTCGCAAATACGAGGCCTTCGCTTCGTTCTCTTTTCATCGTATGGCAGCTTTAATATTTACCGCAA
   PhCRN_01-r710 ATGGAGCTCAATTTACGTATAACGACGAGCAGCTGTGGAGGGGATACGTGAAAGGCGCATTGGAATCGCAAATACGAGGCCTTCGCTTCGTTCTCTTTTCATCGTATGGCAGCTTTAATATTTACCGCAA
   PhCRN_01-r100 ATGAAGCTCAAATTACGTATAACGACGAGCAGCTGTGGAGGGGATACGTGAAAGGCGCATTGGATTCGCAAATACGAGGCCTTCGCTTCGTTCTCTTTTCATCGTATGGCAGCTTTAATATTTACCGCAA
   PhCRN_01-r703 ATGAAGCTCAAATTACGTATAACGACGAGCAGCTGTGGAGGGGATACGTGAAAGGCGCATTGGAATCGCAAATACGAGGCCTTCGCTTCGTTCTCTTTTCATCGTATGGCAGCTTTAATATTTACCGCAA
       Consensus ATGaAGCTCAAaTTACGTATAACGACGAGCAGCTGTGGAGGGGATACGTGAAAGGCGCATTGGAaTCGCAAATACGAGGCCTTCGCTTCGTTCTCTTTTCATCGTATGGCAGCTTTAATATTTACCGCAA

                 911    920       930       940       950       960       970       980       990       1000      1010      1020      1030      1040
                 |--------+---------+---------+---------+---------+---------+---------+---------+---------+---------+---------+---------+---------|
PhCRN_01_cluster ATTTGAACGCGCTGGAACTCCGATCCTTGTGCCTCCTCAGAATACTTTTGGATTGAATGCCACACCATACAAGCCTGGCCTGCAGCTTTCACGTGAGGAGCTGAAAGAAATGGCTCAAAACAGCATTGGC
   PhCRN_01-r304 ATTTGAACGCGCTGGAACTCCGATCCTTGTGCCTCCTCAGAATACTTTTGGATTGAATGCCACACCATACAAGCCTGGCCTGCAGCTTTCACGTGAGGAGCTGAAAGAAATGGCTCAAAACAGCATTGGC
   PhCRN_01-r710 ATTTGAACGCGCTGGAACTCCGATCCTTGTGCCTCCTCAGAATACTTTTGGATTGAATGCCACACCATACAAGCCTGGCCTGCAGCTTTCACGTGAGGAGCTGAAAGAAATGGCTCAAAACAGCATTGGC
   PhCRN_01-r100 ATTTGAACGCGCTGGAACTCCGATCCTTGTGCCTCCTCAGAATACTTTTGGATTGAATGCCACACCATACAAGCCTGGCCTGCAGCTTTCACGTGAGGAGCTGAAAGAAATGGCTCAAAACAGCATTGGC
   PhCRN_01-r703 ATTTGAACGCGCTGGAACTCCGATCCTTGTGCCTCCTCAGAATACTTTTGGATTGAATGCCACACCATACAAGCCTGGCCTGCAGCTTTCACGTGAGGAGCTGAAAGAAATGGCTCAAAACAGCATTGGC
       Consensus ATTTGAACGCGCTGGAACTCCGATCCTTGTGCCTCCTCAGAATACTTTTGGATTGAATGCCACACCATACAAGCCTGGCCTGCAGCTTTCACGTGAGGAGCTGAAAGAAATGGCTCAAAACAGCATTGGC

                 1041   1050      1060      1070      1080      1090      1100      1110      1120      1130      1140      1150      1160      1170
                 |--------+---------+---------+---------+---------+---------+---------+---------+---------+---------+---------+---------+---------|
PhCRN_01_cluster ATATCAGCCTCCGATCTCATTTGGATCTTGTGCTCGGGTCATATCGGAATTGCTCGAGCTATCCTCATCTTTTTGCATCGTAAGTTTGGTTCTGGAAAGCCAAACGATGAAGATGTTGAGCTGGAACTTC
   PhCRN_01-r304 ATATCAGCCTCCGATCTCATTTGGATCTTGTGCTCGGGTCATATCGGAATTGCTCGAGCTATCCTCATCTTTTTGCATCGTAAGTTTGGTTCTGGAAAGCCAAACGATGAAGATGTTGAGCTGGAACTTC
   PhCRN_01-r710 ATATCAGCCTCCGATCTCATTTGGATCTTGTGCTCGGGTCATATCGGAATTGCTCGAGCTATCCTCATCTTTTTGCATCGTAAGTTTGGTTCTGGAAAGCCAAACGATGAAGATGTTGAGCTGGAACTTC
   PhCRN_01-r100 ATATCAGCCTCCGATCTCATTTGGATCTTGTGCTCGGGTCATATCGGAATTGCTCGAGCTATCCTCATCTTTTTGCATCGTAAGTTTGGTTCTGGAAAGCCAAACGATGAAGATGTTGAGCTGGAACTTC
   PhCRN_01-r703 ATATCAGCCTCCGATCTCATTTGGATCTTGTGCTCGGGTCATATCGGAATTGCTCGAGCTATCCTCATCTTTTTGCATCGTAAGTTTGGTTCTGGAAAGCCAAACGATGAAGATGTTGAGCTGGAACTTC
       Consensus ATATCAGCCTCCGATCTCATTTGGATCTTGTGCTCGGGTCATATCGGAATTGCTCGAGCTATCCTCATCTTTTTGCATCGTAAGTTTGGTTCTGGAAAGCCAAACGATGAAGATGTTGAGCTGGAACTTC

                 1171   1180      1190      1200      1210      1220      1230      1240      1250      1260      1270      1280      1290      1300
                 |--------+---------+---------+---------+---------+---------+---------+---------+---------+---------+---------+---------+---------|
PhCRN_01_cluster GCTCCTTGGGGCTGCTGCAAGAAATTCGCTCCAGTTATCGGGGTATCCCAAGTGCTGATGCTTTCGAGCGAGTGAAAGATGCTAATAACCTGCCGGATGAAGCAGTGCTGAAGATGATTGGAATCTTGAA
   PhCRN_01-r304 GCTCCTTGGGGCTGCTGCAAGAAATTCGCTCCAGTTATCGGGGTATCCCAAGTGCTGATGCTTTCGAGCGAGTGAAAGATGCTAATAACCTGCCGGATGAAGCAGTGCTGAAGATGATTGGAATCTTGAA
   PhCRN_01-r710 GCTCCTTGGGGCTGCTGCAAGAAATTCGCTCCAGTTATCGGGGTATCCCAAGTGCTGATGCTTTCGAGCGAGTGAAAGATGCTAATAACCTGCCGGATGAAGCAGTGCTGAAGATGATTGGAATCTTGAA
   PhCRN_01-r100 GCTCCTTGGGGCTGCTGCAAGAAATTCGCTCCAGTTATCGGGGTATCCCAAGTGCTGATGCTTTCGAGCGAGTGAAAGATGCTAATAACCTGCCGGATGAAGCAGTGCTGAAGATGATTGGAATCTTGAA
   PhCRN_01-r703 GCTCCTTGGGGCTGCTGCAAGAAATTCGCTCCAGTTATCGGGGTATCCCAAGTGCTGATGCTTTCGAGCGAGTGAAAGATGCTAATAACCTGCCGGATGAAGCAGTGCTGAAGATGATTGGAATCTTGAA
       Consensus GCTCCTTGGGGCTGCTGCAAGAAATTCGCTCCAGTTATCGGGGTATCCCAAGTGCTGATGCTTTCGAGCGAGTGAAAGATGCTAATAACCTGCCGGATGAAGCAGTGCTGAAGATGATTGGAATCTTGAA

                 1301   1310      1320      1330      1340      1350      1360      1370      1380      1390      1400      1410      1420      1430
                 |--------+---------+---------+---------+---------+---------+---------+---------+---------+---------+---------+---------+---------|
PhCRN_01_cluster TGGTGTTGCTTCTGGCAAGGTTATGCTTGTATCAGATGGACATCGAACTCCTAGAAGCCAGTTTGCTGCGGAATTGTTGACAAGGTACGGGTTCTTGTACGAGGATCAGGCGGGGCAGCTTCAGTTCGCT
   PhCRN_01-r304 TGGTGTTGCTTCTGGCAAGGTTGTGCTTGTATCAGATGGACATCGAACTCCTAGAAGCCAGTTTGCTGCGGAATTGTTGACAAGGTACGGGTTCTTGTACGAGGATCAGGCGGGGCAGCTTCAGTTCGCT
   PhCRN_01-r710 TGGTGTTGCTTCTGGCAAGGTTGTGCTTGTATCAGATGGACATCGAACTCCTAGAAGCCAGTTTGCTGCGGAATTGTTGACAAGGTACGGGTTCTTGTACGAGGATCAGGCGGGGCAGCTTCAGTTCGCT
   PhCRN_01-r100 TGGTGTTGCTTCTGGCAAGGTTGTGCTTGTATCAGATGGACATCGAACTCCTAGAAGCCAGTTTGCTGCGGAATTGTTGACAAGGTACGGGTTCTTGTACGAGGATCAGGCGGGGCAGCTTCAGTTCGCT
   PhCRN_01-r703 TGGTGTTGCTTCTGGCAAGGTTATGCTTGTATCAGATGGACATCGAACTCCTAGAAGCCAGTTTGCTGCGGAATTGTTGACAAGGTACGGGTTCTTGTACGAGGATCAGGCGGGGCAGCTTCAGTTCGCT
       Consensus TGGTGTTGCTTCTGGCAAGGTTgTGCTTGTATCAGATGGACATCGAACTCCTAGAAGCCAGTTTGCTGCGGAATTGTTGACAAGGTACGGGTTCTTGTACGAGGATCAGGCGGGGCAGCTTCAGTTCGCT

                 1431   1440      1450      1460      1470      1480      1490      1500      1510      1520      1530      1540      1550      1560
                 |--------+---------+---------+---------+---------+---------+---------+---------+---------+---------+---------+---------+---------|
PhCRN_01_cluster TCAAGCATGCACCTCAAGATCTGGCTGTATTCAAATCGAACAGATCCAATTTCGTACATGGTATCAGACGTCGCACACGAAGACTTTGTTGTCGCGTGCGTACAGCGAATGAGTGCTTTGCGACTGCAGA
   PhCRN_01-r304 TCAAGCATGCACCTCAAGATCTGGCTGTATTCAAATCGAACAGATCCAATTTCGTACATGGTATCAGACGTCGCACACGAAGACTTTGTTGTCGCGTGCGTACAGCGAATGAGTGCTTTGCGACTGCAGA
   PhCRN_01-r710 TCAAGCATGCACCTCAAGATCTGGCTGTATTCAAATCGAACAGATCCAATTTCGTACATGGTATCAGACGTCGCACACGAAGACTTTGTTGTCGCGTGCGTACAGCGAATGAGTGCTTTGCGACTGCAGA
   PhCRN_01-r100 TCAAGCATGCACCTCAAGATCTGGCTGTATTCAAATCGAACAGATCCAATTTCGTACATGGTATCAGACGTCGCACACGAAGACTTTGTTGTCGCGTGCGTACAGCGAATGAGTGCTTTGCGACTGCAGA
   PhCRN_01-r703 TCAAGCATGCACCTCAAGATCTGGCTGTATTCAAATCGAACAGATCCAATTTCGTACATGGTATCAGACGTCGCACACGAAGACTTTGTTGTCGCGTGCGTACAGCGAATGAGTGCTTTGCGACTGCAGA
       Consensus TCAAGCATGCACCTCAAGATCTGGCTGTATTCAAATCGAACAGATCCAATTTCGTACATGGTATCAGACGTCGCACACGAAGACTTTGTTGTCGCGTGCGTACAGCGAATGAGTGCTTTGCGACTGCAGA

                 1561   1570      1580      1590      1600      1610      1620      1630      1640      1650      1660      1670      1680      1690
                 |--------+---------+---------+---------+---------+---------+---------+---------+---------+---------+---------+---------+---------|
PhCRN_01_cluster ACTTCGCGAACGAAAACACCAACGGCATTGCTCGAGAACGCCAAATACAGATGGAGCTATACGGCGCCACAGTATCATGCTTGCCTCGAGATGTGTTGGTTACCCCTGAATGGCGAACGACTGATGGCAA
   PhCRN_01-r304 ACTTCGCGAACGAAAACACCAACGGCATTGCTCGAGAACGCCAAATACAGATGGAGCTATACGGCGCCACAGTATCATGCTTGCCTCGAGATGTGTTGGTTACCCCTGAATGGCGAACGACTGATGGCAA
   PhCRN_01-r710 ACTTCGCGAACGAAAACACCAACGGCATTGCTCGAGAACGCCAAATACAGATGGAGCTATACGGCGCCACAGTATCATGCTTGCCTCGAGATGTGTTGGTTACCCCTGAATGGCGAACGACTGATGGCAA
   PhCRN_01-r100 ACTTCGCGAACGAAAACACCAACGGCATTGCTCGAGAACGCCAAATACAGATGGAGCTATACGGCGCCACAGTATCATGCTTGCCTCGAGATGTGTTGGTTACCCCTGAATGGCGAACGACTGATGGCAA
   PhCRN_01-r703 ACTTCGCGAACGAAAACACCAACGGCATTGCTCGAGAACGCCAAATACAGATGGAGCTATACGGCGCCACAGTATCATGCTTGCCTCGAGATGTGTTGGTTACCCCTGAATGGCGAACGACTGATGGCAA
       Consensus ACTTCGCGAACGAAAACACCAACGGCATTGCTCGAGAACGCCAAATACAGATGGAGCTATACGGCGCCACAGTATCATGCTTGCCTCGAGATGTGTTGGTTACCCCTGAATGGCGAACGACTGATGGCAA

                 1691   1700      1710      1720      1730      1740      1750      1760      1770      1780      1790      1800      1810      1820
                 |--------+---------+---------+---------+---------+---------+---------+---------+---------+---------+---------+---------+---------|
PhCRN_01_cluster AGGATACATTGATTTAATGATTCGTGGCTCGAGCATTCTTTGGTTTTGGGAGTTGTTAGTGAATGGTGACGATGCTGTCGGCCACTCAAATCGATTTGAAACT-GGGGGGAAGTACCATGGAAGCTTAAC
   PhCRN_01-r304 AGGATACATTGATTTAATGATTCGTGGCTCGAGCATTCTTTGGTTTTGGGAGTTGTTAGTGAATGGTGACGATGCTGTCGGCCACTCAAATCGATTTGAAACT-GGGGGGAAGTACCATGGAAGCTTAAC
   PhCRN_01-r710 AGGATACATTGATTTAATGATTCGTGGCTCGAGCATTCTTTGGTTTTGGGAGTTGTTAGTGAATGGTGACGATGCTGTCGGCCACTCAAATCGATTTGAAACT-GGGGGGAAGTACCATGGAAGCTTAAC
   PhCRN_01-r100 AGGATACATTGATTTAATGATTCGTGGCTCGAGCATTCTTTGGTTTTGGGAGTTGTTAGTGAATGGTGACGATGCTGTCGGCCACTCAAATCGATTTGAAACT-GGGGGGAAGTACCATGGAAGCTTAAC
   PhCRN_01-r703 AGGATACATTGATTTAATGATTCGTGGCTCGAGCATTCTTTGGTTTTGGGAGTTGTTAGTGAATGGTGACGATGCTGTCGGCCACTCAAATCGATTTGAAACTTGGGGGGAAGTACCATGGAAGCTTAAC
       Consensus AGGATACATTGATTTAATGATTCGTGGCTCGAGCATTCTTTGGTTTTGGGAGTTGTTAGTGAATGGTGACGATGCTGTCGGCCACTCAAATCGATTTGAAACT.GGGGGGAAGTACCATGGAAGCTTAAC

                 1821   1830      1840      1850      1860      1870      1880      1890      1900      1910      1920      1930      1940      1950
                 |--------+---------+---------+---------+---------+---------+---------+---------+---------+---------+---------+---------+---------|
PhCRN_01_cluster TGGAAGTTCTAAGTATGTGCTCATCGATTTTCGTCAGAGTAAGGGCGTTCGGCACCAGAAGCATGGGTTTCTGTATGTGTCGTTTGTGGATTCTTACACCAAAGCGCTGGTTTTCAGCACTGGTAAGTCC
   PhCRN_01-r304 TGGAAGTTCTAAGTATGTGCTCATCGATTTTCGTCAGAGTAAGGGCGTTCGGCACCAGAAGCATGGGTTTCTGTATGTGTCGTTTGTGGATTCTTACACCAAAGCGCTGGTTTTCAGCACTGGTAAGTCC
   PhCRN_01-r710 TGGAAGTTCTAAGTATGTGCTCATCGATTTTCGTCAGAGTAAGGGCGTTCGGCACCAGAAGCATGGGTTTCTGTATGTGTCGTTTGTGGATTCTTACACCAAAGCGCTGGTTTTCAGCACTGGTAAGTCC
   PhCRN_01-r100 TGGAAGTTCTAAGTATGTGCTCATCGATTTTCGTCAGAGTAAGGGCGTTCGGCACCAGAAGCATGGGTTTCTGTATGTGTCGTTTGTGGATTCTTACACCAAAGCGCTGGTTTTCAGCACTGGTAAGTCC
   PhCRN_01-r703 TGGAAGTTCTAAGTATGTGCTCATCGATTTTCGTCAGAGTAAGGGCGTTCGGCACCAGAAGCATGGGTTTCTGTATGTGTCGTTTGTGGATTCTTACACCAAAGCGCTGGTTTTCAGCACTGGTAAGTCC
       Consensus TGGAAGTTCTAAGTATGTGCTCATCGATTTTCGTCAGAGTAAGGGCGTTCGGCACCAGAAGCATGGGTTTCTGTATGTGTCGTTTGTGGATTCTTACACCAAAGCGCTGGTTTTCAGCACTGGTAAGTCC

                 1951   1960      1970      1980      1990      2000      2010      2020      2030      2040      2050      2060      2070      2080
                 |--------+---------+---------+---------+---------+---------+---------+---------+---------+---------+---------+---------+---------|
PhCRN_01_cluster GTTGTAAGCGTCAAACTGCTAAGCTAATTAACTGTTCACGTTTAATACATAAGGTTTTATTCTAATAAATATAAAGTTAGTTATATACAAAGATAACGTACACTCCCGAGCAATACCTAGCAAATAAAGC
   PhCRN_01-r304 GTTGTAAGCGTCAAACTGCTAAGCTAATTAACTGTTCACGTTTAATACATAAGGTTTTATTTTAATAAATATAAAGTTAGTTATATACAAAGATGACGTACACTCCCGAGCAATACCTAGCAAATAAAGC
   PhCRN_01-r710 GTTGTAAGCGTCAAACTGCTAAGCTAATTAACTGTTCATGTTTAATACATAAGGTTTTATTCTAATAAATATAAAGTTAGTTATATACAAAGATAG
   PhCRN_01-r100 GTTGTAAGCGTCAAACTGCTAAGCTAATTAACTGTTCACGTTTAATACATAAGGTTTTATT-TAATAAATATAAAGTTAGTTATAAT
   PhCRN_01-r703 GTTGTAAGCGTCAAACTGCTAAGCTAATTAACTGTTCACGTTTAATACATAAGGTTTTTTTTTAATAAATATAAAGTTAGTTATATAC
       Consensus GTTGTAAGCGTCAAACTGCTAAGCTAATTAACTGTTCAcGTTTAATACATAAGGTTTTaTT.TAATAAATATAAAGTTAGTTATAtacaaagat...................................
```

PhCRN_01_AA

```
                  1       10        20        30        40        50        60        70        80        90        100       110       120       130
                  |--------+---------+---------+---------+---------+---------+---------+---------+---------+---------+---------+---------+---------|
PhCRN_01_cluster  FQNLPARGKMVEISLECAIEGQVGSFDVTIDDGKKISALKKAIKAEKPNKIKCDADELRLFLAKCGDAWLTEAEVKGGVPDISSLEELDAVGAPLNVVGLSETDVQFLVTKELAKAKKTRVHVLVVVPEG
    PhCRN_01-r304 FQNLPARGKMVEISLECAIEGQVGSFDVTIDDGKKISALKKAIKAEKPSKIKCDADELRLFLAKCGDAWLTEAEVKGGVPDISSLEELDAVGAPLNVVGLSETDVQFLVTKELAKAKKTRVHVLVVVPEG
    PhCRN_01-r710 FQNLPARGKMVEISLECAIEGQVGSFDVTIDDGKKISALKKAIKAEKPNKIKCDADELRLFLAKCGDAWLTEAEVKGGVPDISSLEELDAVGAPLNVVGLSETDVQFLVTKELAKAKKTRVHVLVVVPEG
    PhCRN_01-r100                                                                                                 VVGLSETDVQFLVTKELAKAKKTRVHVLVVVPEG
    PhCRN_01-r703                                                                                                                                   G
        Consensus fqnlpargkmveislecaiegqvgsfdvtiddgkkisalkkaikaekp.kikcdadelrlflakcgdawlteaevkggvpdissleeldavgaplnvvglsetdvqflvtkelakakktrvhvlvvvpeG

                  131     140       150       160       170       180       190       200       210       220       230       240       250       260
                  |--------+---------+---------+---------+---------+---------+---------+---------+---------+---------+---------+---------+---------|
PhCRN_01_cluster  VSRTEVCDMIRDGRGIRKFNEPLGDSELEYEGGVPATFKTLRKATVAAFASLLDRIPVIFIRAPPLSGKTALCDLLYDHIVRSKPDALVSRILANQMPVDGKFTEFFKFLYGYEFEAFCAHKGDRVVLID
    PhCRN_01-r304 VSRTEVCDMIRDGRGIRKFNEPLGDSELEYEGGVPATFKTLRKATVAAFASLLDRIPVIFIRAPPLSGKTALCDLLYDHIVRSKPDALVSRILANQMPVDGKFTEFFKFLYGYEFEAFCAHKGDRVVLID
    PhCRN_01-r710 VSRTEVCDMIRDGRGIRKFNEPLGDSELEYEGGVPATFKTLRKATVAAFASLLDRIPVIFIRAPPLSGKTALCDLLYDHIVRSKPDALVSRILANQMPVDGKFTEFFKFLYGYEFEAFCAHKGDRVVLID
    PhCRN_01-r100 ASRTEVCDMIRDGRGIRKFNEPLGDSELEYEGGVPATFKTLRKATVAAFASLLDRIPVIFIRAPPLSGKTALCDLLYDHIVRSKPDALVSRILANQMPVDGKFTEFFKFLYGYEFEAFCAHKGDRVVLID
    PhCRN_01-r703 VSRTEVCDMIRDGRGIRKFNEPLGDSELEYEGGVPATFKTLRKATVAAFASLLDRIPVIFIRAPPLSGKTALCDLLYDHIVRSKPDALVSRILANQMPVDGKFTEFFKFLYGYEFEAFCAHKGDRVVLID
        Consensus vSRTEVCDMIRDGRGIRKFNEPLGDSELEYEGGVPATFKTLRKATVAAFASLLDRIPVIFIRAPPLSGKTALCDLLYDHIVRSKPDALVSRILANQMPVDGKFTEFFKFLYGYEFEAFCAHKGDRVVLID

                  261     270       280       290       300       310       320       330       340       350       360       370       380       390
                  |--------+---------+---------+---------+---------+---------+---------+---------+---------+---------+---------+---------+---------|
PhCRN_01_cluster  EAQITYNDEQLWRGYVKGALESQIRGLRFVLFSSYGSFNIYRKFERAGTPILVPPQNTFGLNATPYKPGLQLSREELKEMAQNSIGISASDLIWILCSGHIGIARAILIFLHRKFGSGKPNDEDVELELR
    PhCRN_01-r304 GAQFTYNDEQLWRGYVKGALDSQIRGLRFVLFSSYGSFNIYRKFERAGTPILVPPQNTFGLNATPYKPGLQLSREELKEMAQNSIGISASDLIWILCSGHIGIARAILIFLHRKFGSGKPNDEDVELELR
    PhCRN_01-r710 GAQFTYNDEQLWRGYVKGALESQIRGLRFVLFSSYGSFNIYRKFERAGTPILVPPQNTFGLNATPYKPGLQLSREELKEMAQNSIGISASDLIWILCSGHIGIARAILIFLHRKFGSGKPNDEDVELELR
    PhCRN_01-r100 EAQITYNDEQLWRGYVKGALDSQIRGLRFVLFSSYGSFNIYRKFERAGTPILVPPQNTFGLNATPYKPGLQLSREELKEMAQNSIGISASDLIWILCSGHIGIARAILIFLHRKFGSGKPNDEDVELELR
    PhCRN_01-r703 EAQITYNDEQLWRGYVKGALESQIRGLRFVLFSSYGSFNIYRKFERAGTPILVPPQNTFGLNATPYKPGLQLSREELKEMAQNSIGISASDLIWILCSGHIGIARAILIFLHRKFGSGKPNDEDVELELR
        Consensus eAQiTYNDEQLWRGYVKGAL#SQIRGLRFVLFSSYGSFNIYRKFERAGTPILVPPQNTFGLNATPYKPGLQLSREELKEMAQNSIGISASDLIWILCSGHIGIARAILIFLHRKFGSGKPNDEDVELELR

                  391     400       410       420       430       440       450       460       470       480       490       500       510       520
                  |--------+---------+---------+---------+---------+---------+---------+---------+---------+---------+---------+---------+---------|
PhCRN_01_cluster  SLGLLQEIRSSYRGIPSADAFERVKDANNLPDEAVLKMIGILNGVASGKVMLVSDGHRTPRSQFAAELLTRYGFLYEDQAGQLQFASSMHLKIWLYSNRTDPISYMVSDVAHEDFVVACVQRMSALRLQN
    PhCRN_01-r304 SLGLLQEIRSSYRGIPSADAFERVKDANNLPDEAVLKMIGILNGVASGKVVLVSDGHRTPRSQFAAELLTRYGFLYEDQAGQLQFASSMHLKIWLYSNRTDPISYMVSDVAHEDFVVACVQRMSALRLQN
    PhCRN_01-r710 SLGLLQEIRSSYRGIPSADAFERVKDANNLPDEAVLKMIGILNGVASGKVVLVSDGHRTPRSQFAAELLTRYGFLYEDQAGQLQFASSMHLKIWLYSNRTDPISYMVSDVAHEDFVVACVQRMSALRLQN
    PhCRN_01-r100 SLGLLQEIRSSYRGIPSADAFERVKDANNLPDEAVLKMIGILNGVASGKVVLVSDGHRTPRSQFAAELLTRYGFLYEDQAGQLQFASSMHLKIWLYSNRTDPISYMVSDVAHEDFVVACVQRMSALRLQN
    PhCRN_01-r703 SLGLLQEIRSSYRGIPSADAFERVKDANNLPDEAVLKMIGILNGVASGKVMLVSDGHRTPRSQFAAELLTRYGFLYEDQAGQLQFASSMHLKIWLYSNRTDPISYMVSDVAHEDFVVACVQRMSALRLQN
        Consensus SLGLLQEIRSSYRGIPSADAFERVKDANNLPDEAVLKMIGILNGVASGKVvLVSDGHRTPRSQFAAELLTRYGFLYEDQAGQLQFASSMHLKIWLYSNRTDPISYMVSDVAHEDFVVACVQRMSALRLQN

                  521     530       540       550       560       570       580       590       600       610       620       630       640    647
                  |--------+---------+---------+---------+---------+---------+---------+---------+---------+---------+---------+---------+------|
PhCRN_01_cluster  FANENTNGIARERQIQMELYGATVSCLPRDVLVTPEWRTTDGKGYIDLMIRGSSILWFWELLVNGDDAVGHSNRFETGGKYHGSLTGSSKYVLIDFRQSKGVRHQKHGFLYVSFVDSYTKALVFSTG
    PhCRN_01-r304 FANENTNGIARERQIQMELYGATVSCLPRDVLVTPEWRTTDGKGYIDLMIRGSSILWFWELLVNGDDAVGHSNRFETGGKYHGSLTGSSKYVLIDFRQSKGVRHQKHGFLYVSFVDSYTKALVFSTG
    PhCRN_01-r710 FANENTNGIARERQIQMELYGATVSCLPRDVLVTPEWRTTDGKGYIDLMIRGSSILWFWELLVNGDDAVGHSNRFETGGKYHGSLTGSSKYVLIDFRQSKGVRHQKHGFLYVSFVDSYTKALVFSTG
    PhCRN_01-r100 FANENTNGIARERQIQMELYGATVSCLPRDVLVTPEWRTTDGKGYIDLMIRGSSILWFWELLVNGDDAVGHSNRFETGGKYHGSLTGSSKYVLIDFRQSKGVRHQKHGFLYVSFVDSYTKALVFSTG
    PhCRN_01-r703 FANENTNGIARERQIQMELYGATVSCLPRDVLVTPEWRTTDGKGYIDLMIRGSSILWFWELLVNGDDAVGHSNRFETWGE
        Consensus FANENTNGIARERQIQMELYGATVSCLPRDVLVTPEWRTTDGKGYIDLMIRGSSILWFWELLVNGDDAVGHSNRFETgGkyhgsltgsskyvlidfrqskgvrhqkhgflyvsfvdsytkalvfstg
```

PhCRN_03_NA

```
               1        10        20        30        40        50        60        70        80        90        100       110       120       130
               |--------+---------+---------+---------+---------+---------+---------+---------+---------+---------+---------+---------+---------|
     PhCRN_03   TTCCAGAATCTGCCGGCTCGAGGAAAGATGGTTGAAA-TCTCACTGGAGTGTGCAATCGAAGGCCAGGTAGGCTCGTTCGAAGTGACAATCGACGACTGCAAGAAGATTTCTGCGTTGAAGAAGGCGAT
PhCRN_03-r100  ATTCCAGAATCTGCCGGCTCGAGGAAAGATGGTTGAAAATCTCACTGGAGTGTGCAATCGAAGGCCAGGTAGGCTCGTTCGAAGTGACAATCGACGACTGCAAGAAGATTTCTGCGTTGAAGAAGGCGAT
PhCRN_03-r304  ATTCCAGAATCTGCCGGCTCGAGGAAAGATGGTTGAAAATCTCACTGGAGTGTGCAATCGAAGGCCAGGTAGGCTCGTTCGAAGTGACAATCGACGACTGCAAGAAGATTTCTGCGTTGAAGAAGGCGAT
PhCRN_03-r710  ATTCCAGAATCTGCCGGCTCGAGGAAAGATGGTTGAAA-TCTCACTGGAGTGTGCAATCGAAGGCCAGGTAGGCTCGTTCGAAGTGACAATCGACGACTGCAAGAAGATTTCTGCGTTGAAGAAGGCGAT
PhCRN_03-r703                                                                                                                          GGCGAT
    Consensus  attccagaatctgccggctcgaggaaagatggttgaaa.tctcactggagtgtgcaatcgaaggccaggtaggctcgttcgaagtgacaatcgacgactgcaagaagatttctgcgttgaagaaGGCGAT

               131      140       150       160       170       180       190       200       210       220       230       240       250       260
               |--------+---------+---------+---------+---------+---------+---------+---------+---------+---------+---------+---------+---------|
     PhCRN_03  CAAGGAGGAGATCGACTACTCAGGTCGAGCCAAGGACCTGGAGCTCTCTCTGGCGAAGAAGGGCGAAGGATGGCTACCAATTGAGGACCTTGCTGCGATAGAAGACGGAGTAGCGGTGCCGGGTTTTGAA
PhCRN_03-r100  CAAGGAGGAGATCGACTACTCAGGTCGAGCCAAGGACCTGGAGCTCTCTCTGGCGAAGAAGGGCGAAGGATGGCTACCAATTGAGGACCTTGCTGCGATAGAAGACGGAGTAGCGGTGCCGGGTTTTGAA
PhCRN_03-r304  CAAGGAGGAGATCGACTACTCAGGTCGAGCCAAGGACCTGGAGCTCTCTCTGGCGAAGAAGGGCGAAGGATGGCTACCAATTGAGGACCTTGCTGCGATAGAAGACGGAGTAGCGGTGCCGGGTTTTGAA
PhCRN_03-r710  CAAGGAGGAGATCGACTACTCAGGTCGAGCCAAGGACCTGGAGCTCTCTCTGGCGAAGAAGGGCGAAGGATGGCTACCAATTGAGGACCTTGCTGCGATAGAAGACGGAGTAGCGGTGCCGGGTTTTGAA
PhCRN_03-r703  CAAGGAGGAGATCGACTACTCAGGTCGAGCCAAGGACCTGGAGCTCTCTCTGGCGAAGAAGGGCGAAGGATGGCTACCAATTGAGGACCTTGCTGCGATAGAAGACGGAGTAGCGGTGCCGGGTTTTGAA
    Consensus  CAAGGAGGAGATCGACTACTCAGGTCGAGCCAAGGACCTGGAGCTCTCTCTGGCGAAGAAGGGCGAAGGATGGCTACCAATTGAGGACCTTGCTGCGATAGAAGACGGAGTAGCGGTGCCGGGTTTTGAA

               261      270       280       290       300       310       320       330       340       350       360       370       380       390
               |--------+---------+---------+---------+---------+---------+---------+---------+---------+---------+---------+---------+---------|
     PhCRN_03  AAGGTGTCATTGGTGGATACAAAGAGTGAGAAGTACTCGGCCTATTCGATTCAGAAAATGTTGCAGATAAAAGGATTGCCCTCACCTCAAACGGAGCAAATTCACGTGCTGGTGGTGGTTCCGAAGGATG
PhCRN_03-r100  AAGGTGTCATTGGTGGATACAAAGAGTGAGAAGTACTCGGCCTATTCGATTCAGAAAATGTTGCAGATAAAAGGATTGCCCTCACCTCAAACGGAGCAAATTCACGTGCTGGTGGTGGTTCCGAAGGATG
PhCRN_03-r304  AAGGTGTCATTGGTGGATACAAAGAGTGAGAAGTACTCGGCCTATTCGATTCAGAAAATGTTGCAGATAAAAGGATTGCCCTCACCTCAAACGGAGCAAATTCACGTGCTGGTGGTGGTTCCGAAGGATG
PhCRN_03-r710  AAGGTGTCATTGGTGGATACAAAGAGTGAGAAGTACTCGGCCTATTCGATTCAGAAAATGTTGCAGATAAAAGGATTGCCCTCACCTCAAACGGAGCAAATTCACGTGCTGGTGGTGGTTCCGAAGGATG
PhCRN_03-r703  AAGGTGTCATTGGTGGATACAAAGAGTGAGAAGTACTCGGCCTATTCGATTCAGAAAATGTTGCAGATAAAAGGATTGCCCTCACCTCAAACGGAGCAAATTCACGTGCTGGTGGTGGTTCCGAAGGATG
    Consensus  AAGGTGTCATTGGTGGATACAAAGAGTGAGAAGTACTCGGCCTATTCGATTCAGAAAATGTTGCAGATAAAAGGATTGCCCTCACCTCAAACGGAGCAAATTCACGTGCTGGTGGTGGTTCCGAAGGATG

               391      400       410       420       430       440       450       460       470       480       490       500       510       520
               |--------+---------+---------+---------+---------+---------+---------+---------+---------+---------+---------+---------+---------|
     PhCRN_03  AAAACGACCGCTCCGCAGCCATGGCTCTCGGCGTGGCACCCTCTTTGCCACCAACTACAATTCATCGACATCCCGAACGTCTTAAGCGATGGGCTGCGATTAACGAAATGATTCGACAGAAGAATCAAGA
PhCRN_03-r100  AAAACGACCGCTCCGCAGCCATGGCTCTCGGCGTGGCACCCTCTTTGCCACCAACTACAATTCATCGACATCCCGAACGTCTTAAGCGATGGGCTGCGATTAACGAAATGATTCGACAGAAGAATCAAGA
PhCRN_03-r304  AAAACGACCGCTCCGCAGCCATGGCTCTCGGCGTGGCACCCTCTTTGCCACCAACTACAATTCATCGACATCCCGAACGTCTTAAGCGATGGGCTGCGATTAACGAAATGATTCGACAGAAGAATCAAGA
PhCRN_03-r710  AAAACGACCGCTCCGCAGCCATGGCTCTCGGCGTGGCACCCTCTTTGCCACCAACTACAATTCATCGACATCCCGAACGTCTTAAGCGATGGGCTGCGATTAACGAAATGATTCGACAGAAGAATCAAGA
PhCRN_03-r703  AAAACGACCGCTCCGCAGCCATGGCTCTCGGCGTGGCACCCTCTTTGCCACCAACTACAATTCATCGACATCCCGAACGTCTTAAGCGATGGGCTGCGATTAACGAAATGATTCGACAGAAGAATCAAGA
    Consensus  AAAACGACCGCTCCGCAGCCATGGCTCTCGGCGTGGCACCCTCTTTGCCACCAACTACAATTCATCGACATCCCGAACGTCTTAAGCGATGGGCTGCGATTAACGAAATGATTCGACAGAAGAATCAAGA

               521      530       540       550       560       570       580       590       600       610       620       630       640       650
               |--------+---------+---------+---------+---------+---------+---------+---------+---------+---------+---------+---------+---------|
     PhCRN_03  AGGAAACGAGAAGACGAGCACTCGAGACACCAACAAGAAGCGAAAGAACAGAGATATCGATAGCTCGATGCCGTATTCAAGCCTGTCGTGGACCGATCTTGAACCGATTCTGTCAATGGAGGACTTTA--
PhCRN_03-r100  AGGAAACGAGAAGACGAGCACTCGAGACACCAACAAGAAGCGAAAGAACAGAGATATCGATAGCTCGATGCCGTATTCAAGCCTGTCGTGGACCGATCTTGAACCGATTCTGTCAATGGAGGACTTTA--
PhCRN_03-r304  AGGAAACGAGAAGACGAGCACTCGAGACACCAACAAGAAGCGAAAGAACAGAGATATCGATAGCTCGATGCCGTATTCAAGCCTGTCGTGGACCGATCTTGAACCGATTCTGTCAATGGAGGACTTTA--
PhCRN_03-r710  AGGAAACGAGAAGACGAGCACTCGAGACACCAACAAGAAGCGAAAGAACAGAGATATCGATAGCTCGATGCCGTATTCAAGCCTGTCGTGGACCGATCTTGAACCGATTCTGTCAATGGAGGACTTTATA
PhCRN_03-r703  AGGAAACGAGAAGACGAGCACTCGAGACACCAACAAGAAGCGAAAGAACAGAGATATCGATAGCTCGATGCCGTATTCAAGCCTGTCGTGGACCGATCTTGAACCGATTCTGTCAATGGAGGACTTTA--
    Consensus  AGGAAACGAGAAGACGAGCACTCGAGACACCAACAAGAAGCGAAAGAACAGAGATATCGATAGCTCGATGCCGTATTCAAGCCTGTCGTGGACCGATCTTGAACCGATTCTGTCAATGGAGGACTTTA..

               651      660       670       680       690       700       710       720       730       740       750       760       770       780
               |--------+---------+---------+---------+---------+---------+---------+---------+---------+---------+---------+---------+---------|
     PhCRN_03  ATCTCGAAGCGAGTGCTGTTCCTCAAAAAGTTGTGGAAGAGCTACGTGATCGGCTTATGCAAGTACGTAAACTGTATGGGGATGTTTACTCGGGAAAGGAAGCCAAGCGTCAAGTCTTTATTATGGCTAT
PhCRN_03-r100  ATCTCGAAGCGAGTGCTGTTCCTCAAAAAGTTGTGGAAGAGCTACGTGATCGGCTTATGCAAGTACGTAAACTGTATGGGGATGTTTACTCGGGAAAGGAAGCCAAGCGTCAAGTCTTTATTATGGCTAT
PhCRN_03-r304  ATCTCGAAGCGAGTGCTGTTCCTCAAAAAGTTGTGGAAGAGCTACGTGATCGGCTTATGCAAGTACGTAAACTGTATGGGGATGTTTACTCGGGAAAGGAAGCCAAGCGTCAAGTCTTTATTATGGCTAT
PhCRN_03-r710  ATCTCGAAGCGAGTGCTGTTCCTCAAAAAGTTGTGGAAGAGCTACGTGATCGGCTTATGCAAGTACGTAAACTGTATGGGGATGTTTACTCGGGAAAGGAAGCCAAGCGTCAAGTCTTTATTATGGCTAT
PhCRN_03-r703  ATCTCGAAGCGAGTGCTGTTCCTCAAAAAGTTGTGGAAGAGCTACGTGATCGGCTTATGCAAGTACGTAAACTGTATGGGGATGTTTACTCGGGAAAGGAAGCCAAGCGTCAAGTCTTTATTATGGCTAT
    Consensus  ATCTCGAAGCGAGTGCTGTTCCTCAAAAAGTTGTGGAAGAGCTACGTGATCGGCTTATGCAAGTACGTAAACTGTATGGGGATGTTTACTCGGGAAAGGAAGCCAAGCGTCAAGTCTTTATTATGGCTAT

               781      790       800       810       820       830       840       850       860       870       880       890       900       910
               |--------+---------+---------+---------+---------+---------+---------+---------+---------+---------+---------+---------+---------|
     PhCRN_03  TATTGAAGCTGTATGCCTTATGCTTGGTGATGCCACGATTCTTGTTGAAGAGGATGTGAAAGGAAAGAACGTACATGTGCACGGTCGCTTCGAGTTTGTGCTGAAGCGTGGCAAGAAGCGTGTATCCATC
PhCRN_03-r100  TATTGAAGCTGTATGCCTTATGCTTGGTGATGCCACGATTCTTGTTGAAGAGGATGTGAAAGGAAAGAACGTACATGTGCACGGTCGCTTCGAGTTTGTGCTGAAGCGTGGCAAGAAGCGTGTATCCATC
PhCRN_03-r304  TATTGAAGCTGTATGCCTTATGCTTGGTGATGCCACGATTCTTGTTGAAGAGGATGTGAAAGGAAAGAACGTACATGTGCACGGTCGCTTCGAGTTTGTGCTGAAGCGTGGCAAGAAGCGTGTATCCATC
PhCRN_03-r710  TATTGAAGCTGTATGCCTTATGCTTGGTGATGCCACGATTCTTGTTGAAGAGGATGTGAAAGGAAAGAACGTACATGTGCACGGTCGCTTCGAGTTTGTGCTGAAGCGTGGCAAGAAGCGTGTATCCATC
PhCRN_03-r703  TATTGAAGCTGTATGCCTTATGCTTGGTGATGCCACGATTCTTGTTGAAGAGGATGTGAAAGGAAAGAACGTACATGTGCACGGTCGCTTCGAGTTTGTGCTGAAGCGTGGCAAGAAGCGTGTATCCATC
    Consensus  TATTGAAGCTGTATGCCTTATGCTTGGTGATGCCACGATTCTTGTTGAAGAGGATGTGAAAGGAAAGAACGTACATGTGCACGGTCGCTTCGAGTTTGTGCTGAAGCGTGGCAAGAAGCGTGTATCCATC

               911      920       930       940       950       960       970       980       990       1000      1010      1020      1030      1040
               |--------+---------+---------+---------+---------+---------+---------+---------+---------+---------+---------+---------+---------|
     PhCRN_03  GTCGAAGCAAAACGCGATGATATCCCGCAAGGTATTGCCCAGAATGTGGCCGGCCTCGAAGCTTTGTCTGACGTGGAGGGATTAGAGCGGACACTTGGTATTGTTACCAACTATCTGGAGTGGGTATTCA
PhCRN_03-r100  GTCGAAGCAAAACGCGATGATATCCCGCAAGGTATTGCCCAGAATGTGGCCGGCCTCGAAGCTTTGTCTGACGTGGAGGGATTAGAGCGGACACTTGGTATTGTTACCAACTATCTGGAGTGGGTATTCA
PhCRN_03-r304  GTCGAAGCAAAACGCGATGATATCCCGCAAGGTATTGCCCAGAATGTGGCCGGCCTCGAAGCTTTGTCTGACGTGGAGGGATTAGAGCGGACACTTGGTATTGTTACCAACTATCTGGAGTGGGTATTCA
PhCRN_03-r710  GTCGAAGCAAAACGCGATGATATCCCGCAAGGTATTGCCCAGAATGTGGCCGGCCTCGAAGCTTTGTCTGACGTGGAGGGATTAGAGCGGACACTTGGTATTGTTACCAACTATCTGGAGTGGGTATTCA
PhCRN_03-r703  GTCGAAGCAAAACGCGATGATATCCCGCAAGGTATTGCCCAGAATGTGGCCGGCCTCGAAGCTTTGTCTGACGTGGAGGGATTAGAGCGGACACTTGGTATTGTTACCAACTATCTGGAGTGGGTATTCA
    Consensus  GTCGAAGCAAAACGCGATGATATCCCGCAAGGTATTGCCCAGAATGTGGCCGGCCTCGAAGCTTTGTCTGACGTGGAGGGATTAGAGCGGACACTTGGTATTGTTACCAACTATCTGGAGTGGGTATTCA

               1041     1050      1060      1070      1080      1090      1100      1110      1120      1130      1140      1150      1160      1170
               |--------+---------+---------+---------+---------+---------+---------+---------+---------+---------+---------+---------+---------|
     PhCRN_03  TCAGCGATGACGATGAGAAAATTCGACGGATGAATACAACGCTCAAAGTCTATGGAGCGGTGCCTTCAACTAAAGAACTTAAGGAAATTGTCGGCATGATTTGTGGTTTGCTCGCGAATAGCACTTAATA
PhCRN_03-r100  TCAGCGATGACGATGAGAAAATTCGACGGATGAATACAACGCTCAAAGTCTATGGAGCGGTGCCTTCAACTAAAGAACTTAGGGAAATTGTCGGCATGATTTGTGGTTTGCTCGCGAATAGCACTTAATA
PhCRN_03-r304  TCAGCGATGACGATGAGAAAATTCGACGGATGAATACAACGCTCAAAGTCTATGGAGCGGTGCCTTCAACTAAAGAACTTAGGGAAATTGTCGGCATGATTTGTGGTTTGCTCGCGAATAGCACTTAATA
PhCRN_03-r710  TCAGCGATGACGATGAGAAAATTCGACGGATGAATACAACGCTCAAAGTCTATGGAGCGGTGCCTTCAACTAAAGAACTTAGGGAAATTGTCGGCATGATTTGTGGTTTGCTCGCGAATAGCACTTAATA
PhCRN_03-r703  TCAGCGATGACGATGAGAAAATTCGACGGATGAATACAACGCTCAAAGTCTATGGAGCGGTGCCTTCAACTAAAGAACTTAAGGAAATTGTCGGCATGATTTGTGGTTTGCTCGCGAATAGCACTTAATA
    Consensus  TCAGCGATGACGATGAGAAAATTCGACGGATGAATACAACGCTCAAAGTCTATGGAGCGGTGCCTTCAACTAAAGAACTTAgGGAAATTGTCGGCATGATTTGTGGTTTGCTCGCGAATAGCACTTAATA

               1171     1180      1190      1200      1210      1220      1230      1240      1250      1260      1270      1280      1290      1300
               |--------+---------+---------+---------+---------+---------+---------+---------+---------+---------+---------+---------+---------|
     PhCRN_03  GTCTCGCAATTACAATATTTTTTACGAATGCCATTCCGTGTTGGAAAGTTGCTTTCATCAGTTTTATTAGACCCGGCCCACTGCAAGTGCTTGCAGTTAACATCGAAGCACAGCACAAAATAATTTTTAT
PhCRN_03-r100  GTCTCGCAATTACAATATTTTTTACGAATGCCATTCCGTGTTGGAAAGTTGCTTTCATCAGTTTTATTAGACCCGGCCCACTGCAAGTGCTTGCAGTTAACATCGAAGCACAGCACAAAATAATTTTTAT
PhCRN_03-r304  GTCTCGCAATTACAATATTTTTTACGAATGCCATTCCGTGTTGGAAAGTTGCTTTCATCAGTTTTATTAGACCCGGCCCACTGCAAGTGCTTGCAGTTAACATCGAAGCACAGCACAAAATAATTTTTAT
PhCRN_03-r710  GTCTCGCAATTACAATATTTTTTACGAATGCCATTCCGTGTTGGAAAGTTGACTTTTAAACG
PhCRN_03-r703  GTCTCGCAATTACAATATTTTTTACGAATGCCATTCCGTGTTGGAAAGTTGCTTTCATCAGTTTTATTAGACCCGGCCCACTGCAAGTGCTTGCAGTTAACATCGAAGCACAGCACAAAATAATTTTTAT
    Consensus  GTCTCGCAATTACAATATTTTTTACGAATGCCATTCCGTGTTGGAAAGTTGctTTcatcAgttttattagacccggcccactgcaagtgcttgcagttaacatcgaagcacagcacaaaataatttttat

               1301     1310      1320      1330     1339
               |--------+---------+---------+--------|
     PhCRN_03  TCCTGATAAATAAAAAGTTTGTTATATAGTTAGAGATGA
PhCRN_03-r100  TCCTGATAAATAAAAAGTTTGTTATATAAGCGA
PhCRN_03-r304  TCCTGATAAATAAAAAGTTTGTTATATAAGCGA
PhCRN_03-r710
PhCRN_03-r703  CCTTGATAAATAAAAAGTTTGTTATATAAAGTTAAAA
    Consensus  tcctgataaataaaaagtttgttatataa..........
```

PhCRN_03_AA

```
                   1        10        20        30        40        50        60        70        80        90        100       110       120       130
                   |--------+---------+---------+---------+---------+---------+---------+---------+---------+---------+---------+---------+---------|
PhCRN_03-r100              RWLKISLECAIEGQVGSFEVTIDDCKKISALKKAIKEEIDYSGRAKDLELSLAKKGEGWLPIEDLAAIEDGVAVPGFEKVSLVDTKSEKYSAYSIQKMLQIKGLPSPQTEQIHVLVVVPKDE
PhCRN_03-r304              RWLKISLECAIEGQVGSFEVTIDDCKKISALKKAIKEEIDYSGRAKDLELSLAKKGEGWLPIEDLAAIEDGVAVPGFEKVSLVDTKSEKYSAYSIQKMLQIKGLPSPQTEQIHVLVVVPKDE
PhCRN_03-r710      FQNLPARGKMVEISLECAIEGQVGSFEVTIDDCKKISALKKAIKEEIDYSGRAKDLELSLAKKGEGWLPIEDLAAIEDGVAVPGFEKVSLVDTKSEKYSAYSIQKMLQIKGLPSPQTEQIHVLVVVPKDE
PhCRN_03_cluster   FQNLPARGKMVEISLECAIEGQVGSFEVTIDDCKKISALKKAIKEEIDYSGRAKDLELSLAKKGEGWLPIEDLAAIEDGVAVPGFEKVSLVDTKSEKYSAYSIQKMLQIKGLPSPQTEQIHVLVVVPKDE
PhCRN_03-r703                                                AIKEEIDYSGRAKDLELSLAKKGEGWLPIEDLAAIEDGVAVPGFEKVSLVDTKSEKYSAYSIQKMLQIKGLPSPQTEQIHVLVVVPKDE
Consensus          ............islecaiegqvgsfevtiddckkisalkkAIKEEIDYSGRAKDLELSLAKKGEGWLPIEDLAAIEDGVAVPGFEKVSLVDTKSEKYSAYSIQKMLQIKGLPSPQTEQIHVLVVVPKDE

                   131      140       150       160       170       180       190       200       210       220       230       240       250       260
                   |--------+---------+---------+---------+---------+---------+---------+---------+---------+---------+---------+---------+---------|
PhCRN_03-r100      NDRSAAMALGVAPSLPPTTIHRHPERLKRWAAINEMIRQKNQEGNEKTSTRDTNKKRKNRDIDSSMPYSSLSWTDLEPILSMEDFNLEASAVPQKVVEELRDRLMQVRKLYGDVYSGKEAKRQVFIMAII
PhCRN_03-r304      NDRSAAMALGVAPSLPPTTIHRHPERLKRWAAINEMIRQKNQEGNEKTSTRDTNKKRKNRDIDSSMPYSSLSWTDLEPILSMEDFNLEASAVPQKVVEELRDRLMQVRKLYGDVYSGKEAKRQVFIMAII
PhCRN_03-r710      NDRSAAMALGVAPSLPPTTIHRHPERLKRWAAINEMIRQKNQEGNEKTSTRDTNKKRKNRDIDSSMPYSSLSWTDLEPILSMEDFNLEASAVPQKVVEELRDRLMQVRKLYGDVYSGKEAKRQVFIMAII
PhCRN_03_cluster   NDRSAAMALGVAPSLPPTTIHRHPERLKRWAAINEMIRQKNQEGNEKTSTRDTNKKRKNRDIDSSMPYSSLSWTDLEPILSMEDFNLEASAVPQKVVEELRDRLMQVRKLYGDVYSGKEAKRQVFIMAII
PhCRN_03-r703      NDRSAAMALGVAPSLPPTTIHRHPERLKRWAAINEMIRQKNQEGNEKTSTRDTNKKRKNRDIDSSMPYSSLSWTDLEPILSMEDFNLEASAVPQKVVEELRDRLMQVRKLYGDVYSGKEAKRQVFIMAII
Consensus          NDRSAAMALGVAPSLPPTTIHRHPERLKRWAAINEMIRQKNQEGNEKTSTRDTNKKRKNRDIDSSMPYSSLSWTDLEPILSMEDFNLEASAVPQKVVEELRDRLMQVRKLYGDVYSGKEAKRQVFIMAII

                   261      270       280       290       300       310       320       330       340       350       360       370       380    387
                   |--------+---------+---------+---------+---------+---------+---------+---------+---------+---------+---------+---------+------|
PhCRN_03-r100      EAVCLMLGDATILVEEDVKGKNVHVHGRFEFVLKRGKKRVSIVEAKRDDIPQGIAQNVAGLEALSDVEGLERTLGIVTNYLEWVFISDDDEKIRRMNTTLKVYGAVPSTKELREIVGMICGLLANST
PhCRN_03-r304      EAVCLMLGDATILVEEDVKGKNVHVHGRFEFVLKRGKKRVSIVEAKRDDIPQGIAQNVAGLEALSDVEGLERTLGIVTNYLEWVFISDDDEKIRRMNTTLKVYGAVPSTKELREIVGMICGLLANST
PhCRN_03-r710      EAVCLMLGDATILVEEDVKGKNVHVHGRFEFVLKRGKKRVSIVEAKRDDIPQGIAQNVAGLEALSDVEGLERTLGIVTNYLEWVFISDDDEKIRRMNTTLKVYGAVPSTKELREIVGMICGLLANST
PhCRN_03_cluster   EAVCLMLGDATILVEEDVKGKNVHVHGRFEFVLKRGKKRVSIVEAKRDDIPQGIAQNVAGLEALSDVEGLERTLGIVTNYLEWVFISDDDEKIRRMNTTLKVYGAVPSTKELKEIVGMICGLLANST
PhCRN_03-r703      EAVCLMLGDATILVEEDVKGKNVHVHGRFEFVLKRGKKRVSIVEAKRDDIPQGIAQNVAGLEALSDVEGLERTLGIVTNYLEWVFISDDDEKIRRMNTTLKVYGAVPSTKELKEIVGMICGLLANST
Consensus          EAVCLMLGDATILVEEDVKGKNVHVHGRFEFVLKRGKKRVSIVEAKRDDIPQGIAQNVAGLEALSDVEGLERTLGIVTNYLEWVFISDDDEKIRRMNTTLKVYGAVPSTKELkEIVGMICGLLANST
```

PhCRN_04_NA

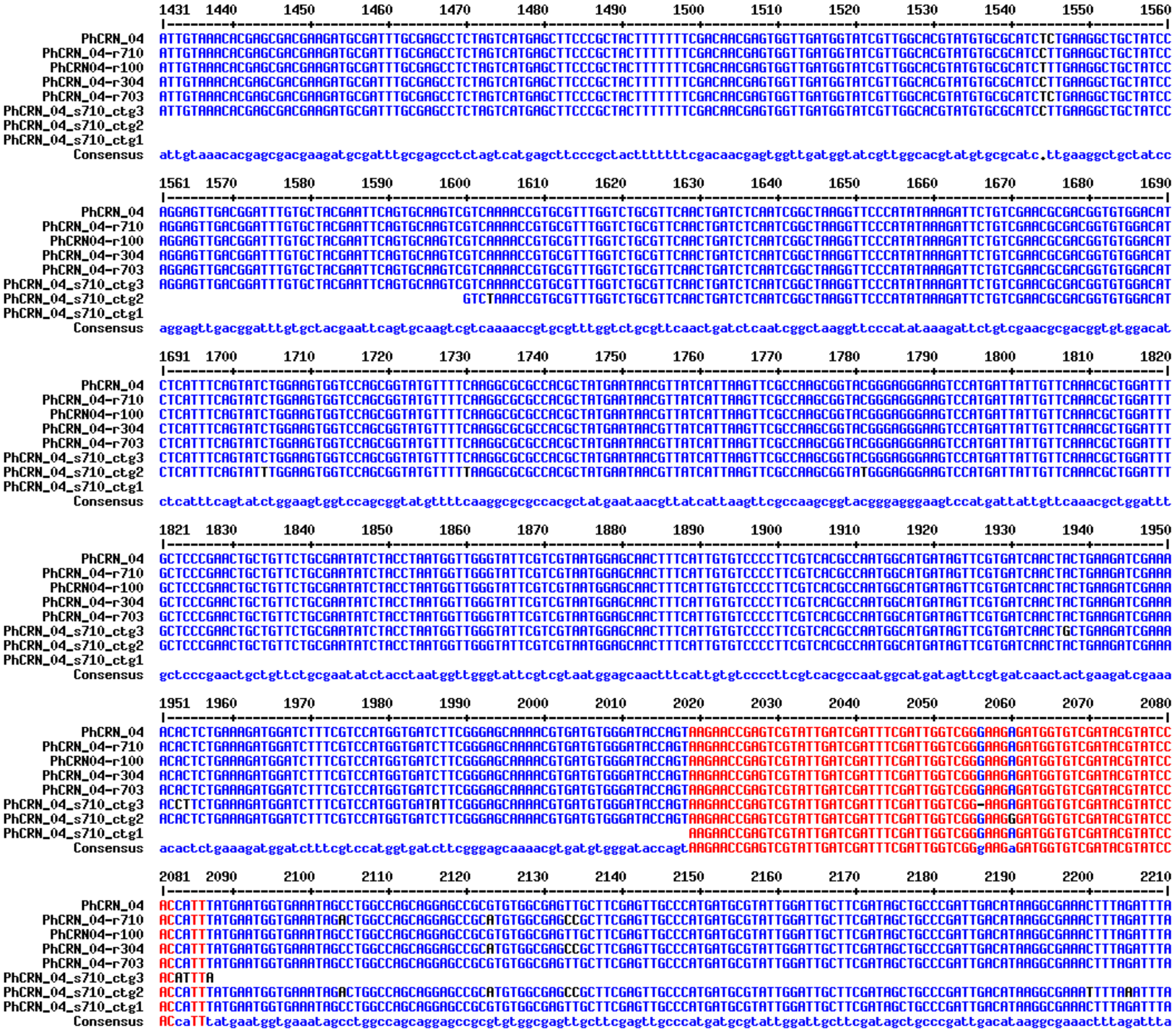

PhCRN_04_AA

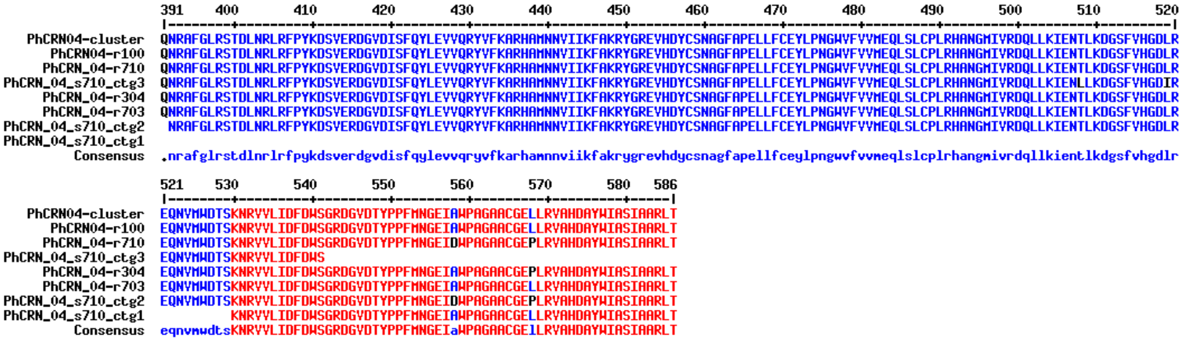

PhCRN_05_NA

```
                   1       10        20        30        40        50        60        70        80        90        100       110       120       130
                   |--------+---------+---------+---------+---------+---------+---------+---------+---------+---------+---------+---------+---------|
         PhCRN_05  ATTGGGGTATTTCCTAGAATTCCGCCGGCTCGAGGAAAGATGGTGAAGCTCTTCTGTGCGA-TTGTTGGTGTGGCGGGAAGCGCGTTCTCGGTGGAAATTGACGAAGGCAAAACGGTGGATGAATTGAAG
PhCRN_05-r304-c1   ATTGGGGTATTTCCTAGAATTCCGCCGGCTCGAGGAAAGATGGTGAAGCTCTTCTGTGCGAATTGTTGGTGTGGCGGGAAGCGCGTTCTCGGTGGAAATTGACGAAGGCAAAACGGTGGATGAATTGAAG
PhCRN_05-r100-c2            GATTCCAGAAT-CCGCCGGCTCGAGGAAAGATGGTGAAGCTCTTCTGTGCGA-TTGTTGGTGTGGCGGGAAGCGCGTTCTCGGTGGAAATTGACGAAGGCAAAACGGTGGATGAATTGAAG
PhCRN_05-r703-uc                                                                                                                                    G
PhCRN_05-r710-c1                                                                                                                                    G
        Consensus  ...........t.c.agaat.ccgccggctcgaggaaagatggtgaagctcttctgtgcga.ttgttggtgtggcgggaagcgcgttctcggtggaaattgacgaaggcaaaacggtggatgaattgaaG

                   131     140       150       160       170       180       190       200       210       220       230       240       250       260
                   |--------+---------+---------+---------+---------+---------+---------+---------+---------+---------+---------+---------+---------|
         PhCRN_05  GGGGCGATCAAGGAGGAGAACTCGGACGACCCAATCCTGAAAAATGTCGCTCCGAAGAACCTGCAGCTCTTTCTGGCGAAGAAAGACGGCGCGTGGCTGAAGTCGAAGGATCCTGCCGTGATTGCTATGC
PhCRN_05-r304-c1   AAGGCGATCAAGGAGGAGAACTCGGACGACCCAATCCTGAAAAATGTCGCTCCGAAGAACCTGCAGCTCTTTCTGGCGAAGAAAGACGGCGCGTGGCTGAAGTCGAAGGATCCTGCCGTGATTGCTATGC
PhCRN_05-r100-c2   AAGGCGATCAAGGAGGAGAACTCGGACGACCCAATCCTGAAAAATGTCGCTCCGAAGAACCTGCAGCTCTTTCTGGCGAAGAAAGACGGCGCGTGGCTGAAGTCGAAGGATCCTGCCGTGATTGCTATGC
PhCRN_05-r703-uc   GGGGCGATCAAGGAGGAGAACTCGGACGACCCAATCCTGAAAAATGTCGCTCCGAAGAACCTGCAGCTCTTTCTGGCGAAGAAAGACGGCGCGTGGCTGAAGTCGAAGGATCCTGCCGTGATTGCTATGC
PhCRN_05-r710-c1   GGGGCGATCAAGGAGGAGAACTCGGACGACCCAATCCTGAAAAATGTCGCTCCGAAGAACCTGCAGCTCTTTCTGGCGAAGAAAGACGGCGCGTGGCTGAAGTCGAAGGATCCTGCCGTGATTGCTATGC
        Consensus  ggGGCGATCAAGGAGGAGAACTCGGACGACCCAATCCTGAAAAATGTCGCTCCGAAGAACCTGCAGCTCTTTCTGGCGAAGAAAGACGGCGCGTGGCTGAAGTCGAAGGATCCTGCCGTGATTGCTATGC

                   261     270       280       290       300       310       320       330       340       350       360       370       380       390
                   |--------+---------+---------+---------+---------+---------+---------+---------+---------+---------+---------+---------+---------|
         PhCRN_05  GGAGTGGGGGTGTTCCCGAGCAAGTCAAGGCACTGCTGGACGTGGAAATGGATCCAGCAGACGAGATTGGTGACTTGTTTGGTGATGCTCCGACGAAGAAGACCATTCATGTGTTGGTGGTGGTTCTAAC
PhCRN_05-r304-c1   GGAGTGGGGGTGTTCCCGAGCAAGTCAAGGCACTGCTGGACGTGGAAATGGATCCAGCAGACGAGATTGGTGACTTGTTTGGTGATGCTCCGACGAAGAAGACCATTCATGTGTTGGTGGTGGTTCTAAC
PhCRN_05-r100-c2   GGAGTGGGGGTGTTCCCGAGCAAGTCAAGGCACTGCTGGACGTGGAAATGGATCCAGCAGACGAGATTGGTGACTTGTTTGGTGATGCTCCGACGAAGAAGACCATTCATGTGTTGGTGGTGGTTCTAAC
PhCRN_05-r703-uc   GGAGTGGGGGTGTTCCCGAGCAAGTCAAGGCACTGCTGGACGTGGAAATGGATCCAGCAGACGAGATTGGTGACTTGTTTGGTGATGCTCCGACGAAGAAGACCATTCATGTGTTGGTGGTGGTTCTAAC
PhCRN_05-r710-c1   GGAGTGGGGGTGTTCCCGAGCAAGTCAAGGCACTGCTGGACGTGGAAATGGATCCAGCAGACGAGATTGGTGACTTGTTTGGTGATGCTCCGACGAAGAAGACCATTCATGTGTTGGTGGTGGTTCTAAC
        Consensus  GGAGTGGGGGTGTTCCCGAGCAAGTCAAGGCACTGCTGGACGTGGAAATGGATCCAGCAGACGAGATTGGTGACTTGTTTGGTGATGCTCCGACGAAGAAGACCATTCATGTGTTGGTGGTGGTTCTAAC

                   391     400       410       420       430       440       450       460       470       480       490       500       510       520
                   |--------+---------+---------+---------+---------+---------+---------+---------+---------+---------+---------+---------+---------|
         PhCRN_05  TCAGCAACAAGCTGCTTCAATCAAGAAGCAACTGCGATACAAGGGAATGAGCACCGAAGCTTCGTGTCGCAAGTTCTTGGATGCGTTGGCGCAGAATCTCGCCACGCTGTACGACTTTGAGTGCAGTTAT
PhCRN_05-r304-c1   TCAGCAACAAGCTGCTTCAATCAAGAAGCAACTGCGATACAAGGGAATGAGCACCGAAGCTTCGTGTCGCAAGTTCTTGGATGCGTTGGCGCAGAATCTCGCCACGCTGTACGACTTTGAGTGCAGTTAT
PhCRN_05-r100-c2   TCAGCAACAAGCTGCTTCAATCAAGAAGCAACTGCGATACAAGGGAATGAGCACCGAAGCTTCGTGTCGCAAGTTCTTGGATGCGTTGGCGCAGAATCTCGCCACGCTGTACGACTTTGAGTGCAGTTAT
PhCRN_05-r703-uc   TCAGCAACAAGCTGCTTCAATCAAGAAGCAACTGCGATACAAGGGAATGAGCACCGAAGCTTCGTGTCGCAAGTTCTTGGATGCGTTGGCGCAGAATCTCGCCACGCTGTACGACTTTGAGTGCAGTTAT
PhCRN_05-r710-c1   TCAGCAACAAGCTGCTTCAATCAAGAAGCAACTGCGATACAAGGGAATGAGCACCGAAGCTTCGTGTCGCAAGTTCTTGGATGCGTTGGCGCAGAATCTCGCCACGCTGTACGACTTTGAGTGCAGTTAT
        Consensus  TCAGCAACAAGCTGCTTCAATCAAGAAGCAACTGCGATACAAGGGAATGAGCACCGAAGCTTCGTGTCGCAAGTTCTTGGATGCGTTGGCGCAGAATCTCGCCACGCTGTACGACTTTGAGTGCAGTTAT

                   521     530       540       550       560       570       580       590       600       610       620       630       640       650
                   |--------+---------+---------+---------+---------+---------+---------+---------+---------+---------+---------+---------+---------|
         PhCRN_05  GGTGATGTCGCGACAATTGGCGACGTCTTCAGTGCTGTCAAAAATGATGAATGGGGTTTTCGTCTGAAGAGAGGGAGGCAGTTGACCAGTGAACCATTGCCAAGCTTCTTCACCGAGGACGAGTGGAAAG
PhCRN_05-r304-c1   GGTGATGTCGCGACAATTGGCGACGTCTTCAGTGCTGTCAAAAATGATGAATGGGGTTTTCGTCTGAAGAGAGGGAGGCAGTTGACCAGTGAACCATTGCCAAGCTTCTTCACCGAGGACGAGTGGAAAG
PhCRN_05-r100-c2   GGTGATGTCGCGACAATTGGCGACGTCTTCAGTGCTGTCAAAAATGATGAATGGGGTTTTCGTCTGAAGAGAGGGAGGCAGTTGACCAGTGAACCATTGCCAAGCTTCTTCACCGAGGACGAGTGGAAAG
PhCRN_05-r703-uc   GGTGATGTCGCGACAATTGGCGACGTCTTCAGTGCTGTCAAAAATGATGAATGGGGTTTTCGTCTGAAGAGAGGGAGGCAGTTGACCAGTGAACCATTGCCAAGCTTCTTCACCGAGGACGAGTGGAAAG
PhCRN_05-r710-c1   GGTGATGTCGCGACAATTGGCGACGTCTTCAGTGCTGTCAAAAATGATGAATGGGGTTTTCGTCTGAAGAGAGGGAGGCAGTTGACCAGTGAACCATTGCCAAGCTTCTTCACCGAGGACGAGTGGAAAG
        Consensus  GGTGATGTCGCGACAATTGGCGACGTCTTCAGTGCTGTCAAAAATGATGAATGGGGTTTTCGTCTGAAGAGAGGGAGGCAGTTGACCAGTGAACCATTGCCAAGCTTCTTCACCGAGGACGAGTGGAAAG

                   651     660       670       680       690       700       710       720       730       740       750       760       770       780
                   |--------+---------+---------+---------+---------+---------+---------+---------+---------+---------+---------+---------+---------|
         PhCRN_05  ACCTCAAGGATTTGAATTGGCGTACAAATCGCCGTATTCACGATGGCAAGGTTCCCACAACCAGCAAAGGGAAATCGTACGTCATTCTCCCGCACGCCATTTTCAGTGATGATCGAGTCGACAGATACAA
PhCRN_05-r304-c1   ACCTCAAGGATTTGAATTGGCGTACAAATCGCCGTATTCACGATGGCAAGGTTCCCACAACCAGCAAAGGGAAATCGTACGTCATTCTCCCGCACGCCATTTTCAGTGATGATCGAGTCGACAGATACAA
PhCRN_05-r100-c2   ACCTCAAGGATTTGAATTGGCGTACAAATCGCCGTATTCACGATGGCAAGGTTCCCACAACCAGCAAAGGGAAATCGTACGTCATTCTCCCGCACGCCATTTTCAGTGATGATCGAGTCGACAGATACAA
PhCRN_05-r703-uc   ACCTCAAGGATTTGAATTGGCGTACAAATCGCCGTATTCACGATGGCAAGGTTCCCACAACCAGCAAAGGGAAATCGTACGTCATTCTCCCGCACGCCATTTTCAGTGATGATCGAGTCGACAGATACAA
PhCRN_05-r710-c1   ACCTCAAGGATTTGAATTGGCGTACAAATCGCCGTATTCACGATGGCAAGGTTCCCACAACCAGCAAAGGGAAATCGTACGTCATTCTCCCGCACGCCATTTTCAGTGATGATCGAGTCGACAGATACAA
        Consensus  ACCTCAAGGATTTGAATTGGCGTACAAATCGCCGTATTCACGATGGCAAGGTTCCCACAACCAGCAAAGGGAAATCGTACGTCATTCTCCCGCACGCCATTTTCAGTGATGATCGAGTCGACAGATACAA

                   781     790       800       810       820       830       840       850       860       870       880       890       900       910
                   |--------+---------+---------+---------+---------+---------+---------+---------+---------+---------+---------+---------+---------|
         PhCRN_05  AACTATTGCGACTAGAGCAAGCGTGGTGTTCGAAGCGACCGAGTTCGAAGTGAAAGATGAAGATGAGTTCTCGGGTAGTTCTCGTTCAAGTAGTGGTCGTTCGGATATTGCATGATTTCATCGTAGTTCT
PhCRN_05-r304-c1   AACTATTGCGACTAGAGCAAGCGTGGTGTTCGAAGCGACCGATTTCGAAGTGAAAGATGAAGATGAGTTCTCGGGTAGTTCTCGTTCAAGTAGTGGTCGTTCGGATATTGCATGATTTCATCGTAGTTCT
PhCRN_05-r100-c2   AACTATTGCGACTAGAGCAAGCGTGGTGTTCGAAGCGACCGATTTCGAAGTGAAAGATGAAGATGAGTTCTCGGGTAGTTCTCGTTCAAGTAGTGGTCGTTCGGATATTGCATGATTTCATCGTAGTTCT
PhCRN_05-r703-uc   AACTATTGCGACTAGAGCAAGCGTGGTGTTCGAAGCGACCGAGTTCGAAGTGAAAGATGAAGATGAGTTCTCGGGTAGTTCTCGTTCAAGTAGTGGTCGTTCGGATATTGCATGATTTCATCGTAGTTCT
PhCRN_05-r710-c1   AACTATTGCGACTAGAGCAAGCGTGGTGTTCGAAGCGACCGAGTTCGAAGTGAAAGATGAAGATGAGTTCTCGGGTAGTTCTCGTTCAAGTAGTGGTCGTTCGGATATTGCATGATTTCATCGTAGTTCT
        Consensus  AACTATTGCGACTAGAGCAAGCGTGGTGTTCGAAGCGACCGAgTTCGAAGTGAAAGATGAAGATGAGTTCTCGGGTAGTTCTCGTTCAAGTAGTGGTCGTTCGGATATTGCATGATTTCATCGTAGTTCT

                   911     920       930       940       950       960       970       980       990       1000      1010      1020      1030      1040
                   |--------+---------+---------+---------+---------+---------+---------+---------+---------+---------+---------+---------+---------|
         PhCRN_05  AGCTGGTACATAAACCTGAATCCAAGCTCAACTACAATTTTTGTTTTGATAACATGTCAACAATCTCCATTACATGACTACTACCATTGCTTCTATGGCTCACTATAGACAATAGCAGAGGCATAGCTTA
PhCRN_05-r304-c1   AGCTGGTACATAAACCTGAATCCAAGCTCAACTACAATTTTTGTTTTTGATAAAT
PhCRN_05-r100-c2   AGCTGGTACATAAACCTGAATCCAAGCTCAACTACTAATTTTTGTTTT
PhCRN_05-r703-uc   AGCTGGTACATAAACCTGAATCCAAGCTCAACTACAATTTTTGTTTTTG
PhCRN_05-r710-c1   AGCTGGTACATAAACCTGAATCCAAGCTCAACTACAATTTTTGTTTTGAAT
        Consensus  AGCTGGTACATAAACCTGAATCCAAGCTCAACTACaAtTTTTgtTTTt..................................................................................
```

PhCRN_05_AA

```
                   1       10        20        30        40        50        60        70        80        90        100       110       120       130
                   |--------+---------+---------+---------+---------+---------+---------+---------+---------+---------+---------+---------+---------|
PhCRN_05-cluster   PPARGKMVKLFCAIVGVAGSAFSVEIDEGKTVDELKGAIKEENSDDPILKNVAPKNLQLFLAKKDGAWLKSKDPAVIAMRSGGVPEQVKALLDVEMDPADEIGDLFGDAPTKKTIHVLVVVLTQQQAASI
PhCRN_05-r100-c2   PPARGKMVKLFCAIVGVAGSAFSVEIDEGKTVDELKKAIKEENSDDPILKNVAPKNLQLFLAKKDGAWLKSKDPAVIAMRSGGVPEQVKALLDVEMDPADEIGDLFGDAPTKKTIHVLVVVLTQQQAASI
PhCRN_05-r304-c1   PPARGKMVKLFCAIVGVAGSAFSVEIDEGKTVDELKKAIKEENSDDPILKNVAPKNLQLFLAKKDGAWLKSKDPAVIAMRSGGVPEQVKALLDVEMDPADEIGDLFGDAPTKKTIHVLVVVLTQQQAASI
    PhCRN_05-r703                                      GAIKEENSDDPILKNVAPKNLQLFLAKKDGAWLKSKDPAVIAMRSGGVPEQVKALLDVEMDPADEIGDLFGDAPTKKTIHVLVVVLTQQQAASI
PhCRN_05-r710-c1                                       GAIKEENSDDPILKNVAPKNLQLFLAKKDGAWLKSKDPAVIAMRSGGVPEQVKALLDVEMDPADEIGDLFGDAPTKKTIHVLVVVLTQQQAASI
        Consensus  ....................................gAIKEENSDDPILKNVAPKNLQLFLAKKDGAWLKSKDPAVIAMRSGGVPEQVKALLDVEMDPADEIGDLFGDAPTKKTIHVLVVVLTQQQAASI

                   131     140       150       160       170       180       190       200       210       220       230       240       250       260
                   |--------+---------+---------+---------+---------+---------+---------+---------+---------+---------+---------+---------+---------|
PhCRN_05-cluster   KKQLRYKGMSTEASCRKFLDALAQNLATLYDFECSYGDVATIGDVFSAVKNDEWGFRLKRGRQLTSEPLPSFFTEDEWKDLKDLNWRTNRRIHDGKVPTTSKGKSYVILPHAIFSDDRVDRYKTIATRAS
PhCRN_05-r100-c2   KKQLRYKGMSTEASCRKFLDALAQNLATLYDFECSYGDVATIGDVFSAVKNDEWGFRLKRGRQLTSEPLPSFFTEDEWKDLKDLNWRTNRRIHDGKVPTTSKGKSYVILPHAIFSDDRVDRYKTIATRAS
PhCRN_05-r304-c1   KKQLRYKGMSTEASCRKFLDALAQNLATLYDFECSYGDVATIGDVFSAVKNDEWGFRLKRGRQLTSEPLPSFFTEDEWKDLKDLNWRTNRRIHDGKVPTTSKGKSYVILPHAIFSDDRVDRYKTIATRAS
    PhCRN_05-r703  KKQLRYKGMSTEASCRKFLDALAQNLATLYDFECSYGDVATIGDVFSAVKNDEWGFRLKRGRQLTSEPLPSFFTEDEWKDLKDLNWRTNRRIHDGKVPTTSKGKSYVILPHAIFSDDRVDRYKTIATRAS
PhCRN_05-r710-c1   KKQLRYKGMSTEASCRKFLDALAQNLATLYDFECSYGDVATIGDVFSAVKNDEWGFRLKRGRQLTSEPLPSFFTEDEWKDLKDLNWRTNRRIHDGKVPTTSKGKSYVILPHAIFSDDRVDRYKTIATRAS
        Consensus  KKQLRYKGMSTEASCRKFLDALAQNLATLYDFECSYGDVATIGDVFSAVKNDEWGFRLKRGRQLTSEPLPSFFTEDEWKDLKDLNWRTNRRIHDGKVPTTSKGKSYVILPHAIFSDDRVDRYKTIATRAS

                   261     270       280       290
                   |--------+---------+---------|
PhCRN_05-cluster   VVFEATEFEVKDEDEFSGSSRSSSGRSDIA
PhCRN_05-r100-c2   VVFEATDFEVKDEDEFSGSSRSSSGRSDIA
PhCRN_05-r304-c1   VVFEATDFEVKDEDEFSGSSRSSSGRSDIA
    PhCRN_05-r703  VVFEATEFEVKDEDEFSGSSRSSSGRSDIA
PhCRN_05-r710-c1   VVFEATEFEVKDEDEFSGSSRSSSGRSDIA
        Consensus  VVFEAT#FEVKDEDEFSGSSRSSSGRSDIA
```

PhCRN_06_NA

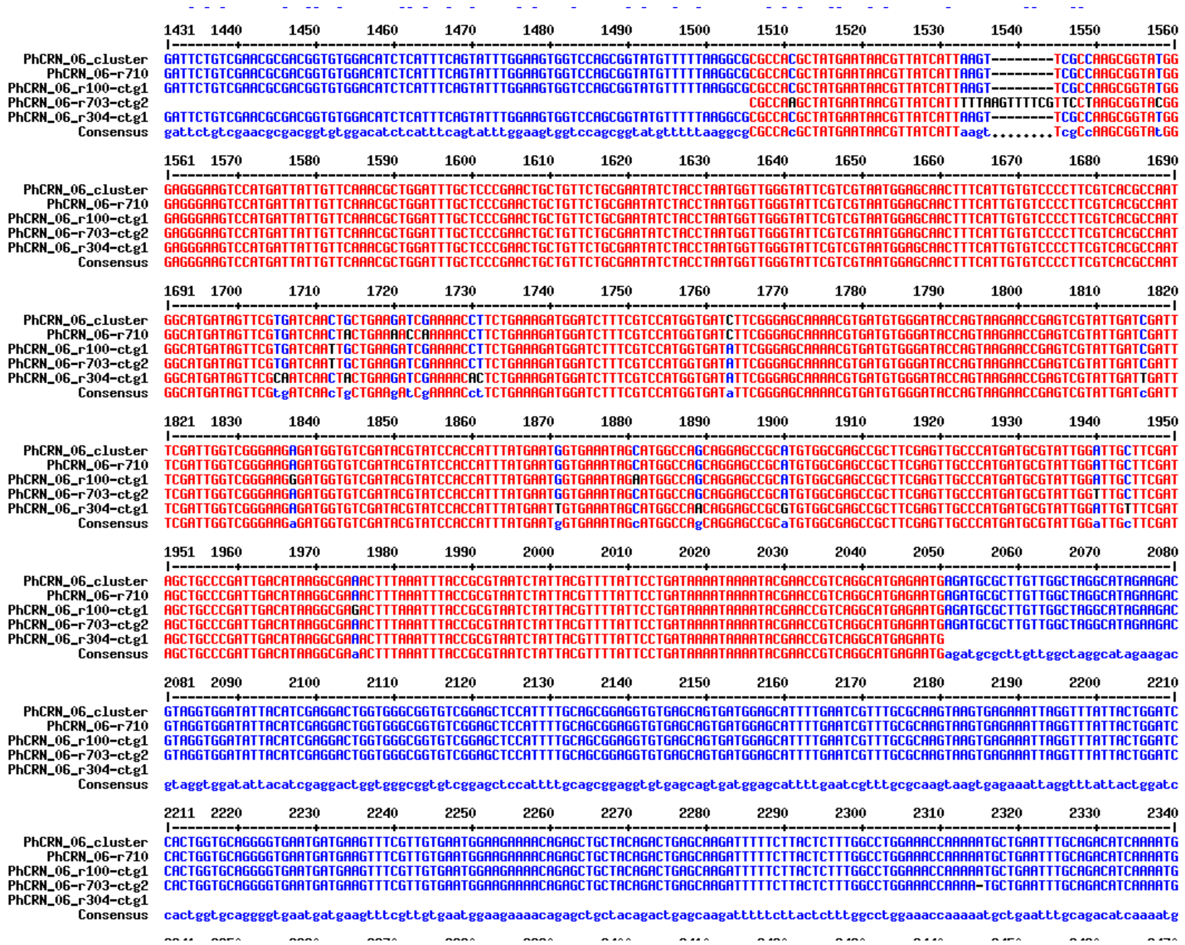

PhCRN_06_AA

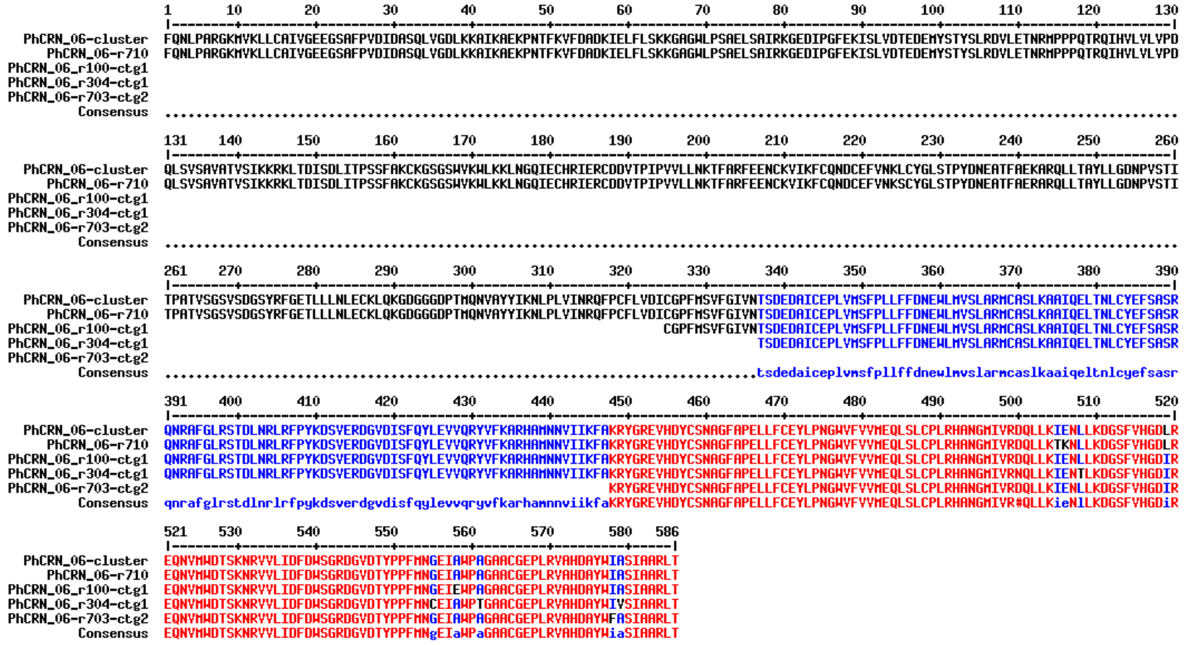

PhCRN_07_NA

```
                391      400       410       420       430       440       450       460       470       480       490       500       510       520
                |--------+---------+---------+---------+---------+---------+---------+---------+---------+---------+---------+---------+---------|
       PhCRN_07 GTGAAAACGACCGCTTCGCAGCCATGGCAATCGGCGTGGCACCCTCTTTGCCACCAACTACAATACACCGGCACCCCGAACGTCTCAAACGATGGGCTGCGATCAATGCAATGATTCGTCAGAAGAATCA
   PhCRN_07-r710 GTGAAAACGACCGCTTCGCAGCCATGGCAATCGGCGTGGCACCCTCTTTGCCACCAACTACAATACACCGGCACCCCGAACGTCTCAAACGATGGGCTGCGATCAATGCAATGATTCGTCAGAAGAATCA
   PhCRN_07-r304 GTGAAAACGACCGCTTCGCAGCCATGGCAATCGGCGTGGCACCCTCTTTGCCACCAACTACAATACACCGGCACCCCGAACGTCTCAAACGATGGGCTGCGATCAATGCAATGATTCGTCAGAAGAATCA
   PhCRN_07-r100 GTGAAAACGACCGCTTCGCAGCCATGGCAATCGGCGTGGCACCCTCTTTGCCACCAACTACAATACACCGGCACCCCGAACGTCTCAAACGATGGGCTGCGATCAATGCAATGATTCGTCAGAAGAATCA
   PhCRN_07-r703
       Consensus gtgaaaacgaccgcttcgcagccatggcaatcggcgtggcaccctctttgccaccaactacaatacaccggcaccccgaacgtctcaaacgatgggctgcgatcaatgcaatgattcgtcagaagaatca

                521      530       540       550       560       570       580       590       600       610       620       630       640       650
                |--------+---------+---------+---------+---------+---------+---------+---------+---------+---------+---------+---------+---------|
       PhCRN_07 AGAGGCGAACCAGAAGACCACGAGTGAAGACGCCAAGAAAACAAACAAAAAGCGGAAAAATCACGATGTCGACAAGACTGTGGGCTGTTCGAGTCTTTGTTGGGAAGATATCAAACCTATTTACCACTTC
   PhCRN_07-r710 AGAGGCGAACCAGAAGACCACGAGTGAAGACGCCAAGAAAACAAGCAAAAAGCGGAAAAATCACGATGTCGACAAGACTGTGGGCTGTTCGAGTCTTTGTTGGGAAGATATCAAACCTATTTACCACTTC
   PhCRN_07-r304 AGAGGCGAACCAGAAGACCACGAGTGAAGACGCCAAGAAAACAAACAAAAAGCGGAAAAATCACGATGTCGACAAGACTGTGGGCTGTTCGAGTCTTTGTTGGGAAGATATCAAACCTATTTACCACTTC
   PhCRN_07-r100 AGAGGCGAACCAGAAGACCACGAGTGAAGACGCCAAGAAAACAAACAAAAAGCGGAAAAATCACGATGTCGACAAGACTGTGGGCTGTTCGAGTCTTTGTTGGGAAGATATCAAACCTATTTACCACTTC
   PhCRN_07-r703                 ACCACGAGTGAAGACGCCAAGAAAACAAGCAAAAAGCGGAAAAATCACGATGTCGACAAGACTGTGGGCTGTTCGAGTCTTTGTTGGGAAGATATCAAACCTATTTACCACTTC
       Consensus agaggcgaaccagaagACCACGAGTGAAGACGCCAAGAAAACAAaCAAAAAGCGGAAAAATCACGATGTCGACAAGACTGTGGGCTGTTCGAGTCTTTGTTGGGAAGATATCAAACCTATTTACCACTTC

                651      660       670       680       690       700       710       720       730       740       750       760       770       780
                |--------+---------+---------+---------+---------+---------+---------+---------+---------+---------+---------+---------+---------|
       PhCRN_07 GACGATTCTTTCGACCTTCAACCAAGCGATATACCTGATGCCGACATCGAGCTACTCCTCGCTCGTATTCGTGACTTGCGGGAACTCTATGGACAAATTTCGGATGGGAAAGAAGCCAAGAGATTGTTTT
   PhCRN_07-r710 GACGATTCTTTCGACCTTCAACCAAGCGATATACCTGATGCCGACATCGAGCTACTCCTCGCTCGTATTCGTGACTTGCGGGAACTCTATGGACAAATTTCGGATGGGAAAGAAGCCAAGAGATTGTTTT
   PhCRN_07-r304 GACGATTCTTTCGACCTTCAACCAAGCGATATACCTGATGCCGACATCGAGCTACTCCTCGCTCGTATTCGTGACTTGCGGGAACTCTATGGACAAATTTCGGATGGGAAAGAAGCCAAGAGATTGTTTT
   PhCRN_07-r100 GACGATTCTTTCGACCTTCAACCAAGCGATATACCTGATGCCGACATCGAGCTACTCCTCGCTCGTATTCGTGACTTGCGGGAACTCTATGGACAAATTTCGGATGGGAAAGAAGCCAAGAGATTGTTTT
   PhCRN_07-r703 GACGATTCTTTCGACCTTCAACCAAGCGATATACCTGATGCCGACATCGAGCTACTCCTCGCTCGTATTCGTGACTTGCGGGAACTCTATGGACAAATTTCGGATGGGAAAGAAGCCAAGAGATGGTTTT
       Consensus GACGATTCTTTCGACCTTCAACCAAGCGATATACCTGATGCCGACATCGAGCTACTCCTCGCTCGTATTCGTGACTTGCGGGAACTCTATGGACAAATTTCGGATGGGAAAGAAGCCAAGAGATtGTTTT

                781      790       800       810       820       830       840       850       860       870       880       890       900       910
                |--------+---------+---------+---------+---------+---------+---------+---------+---------+---------+---------+---------+---------|
       PhCRN_07 TTATCGCCCCGATCCTTGAGACAGTCAGTCGTCTGCTCAAGAACGTCCGCATATTAGTCGAAGAAGACGTGGTTGGGAAGAACGTGCTCCTGAAAGGTCGATTCGAGTTCGTGCTCAAGCGTGGCACCAA
   PhCRN_07-r710 TTATCGCCCCGATCCTTGAGACAGTCAGTCGTCTGCTCAAGAACGTCCGCATATTAGTCGAAGAAGACGTGGTTGGGAAGAACGTGCTCCTGAAAGGTCGATTCGAGTTCGTGCTCAAGCGTGGCACCAA
   PhCRN_07-r304 TTATCGCCCCGATCCTTGAGACAGTCAGTCGTCTGCTCAAGAACGTCCGCATATTAGTCGAAGAAGACGTGGTTGGGAAGAACGTGCTCCTGAAAGGTCGATTCGAGTTCGTGCTCAAGCGTGGCACCAA
   PhCRN_07-r100 TTATCGCCCCGATCCTTGAGACAGTCAGTCGTCTGCTCAAGAACGTCCGCATATTAGTCGAAGAAGACGTGGTTGGGAAGAACGTGCTCCTGAAAGGTCGATTCGAGTTCGTGCTCAAGCGTGGCACCAA
   PhCRN_07-r703 TTATCGCCCCGATCCTTGAGACAGTCAGTCGTCTGCTCAAGAACGTCCGCATATTAGTCGAAGAAGACGTGGTTGGGAAGAACGTGCTCCTGAAAGGTCGATTCGAGTTCGTGCTCAAGCGTGGCACCAA
       Consensus TTATCGCCCCGATCCTTGAGACAGTCAGTCGTCTGCTCAAGAACGTCCGCATATTAGTCGAAGAAGACGTGGTTGGGAAGAACGTGCTCCTGAAAGGTCGATTCGAGTTCGTGCTCAAGCGTGGCACCAA

                911      920       930       940       950       960       970       980       990       1000      1010      1020      1030      1040
                |--------+---------+---------+---------+---------+---------+---------+---------+---------+---------+---------+---------+---------|
       PhCRN_07 GAGAATTTCACTTGTCGAAGCAAAGCGTGAAGATATGCTGCAAGGAATGGTTCAGAATGTGACGGGGCTCGAAGCTCTTGCTGATGTCGAAGATCTTACTGTGACGTATGGCATTGTGACGAATTTTCTC
   PhCRN_07-r710 GAGAATTTCACTTGTCGAAGCAAAGCGTGAAGATATGCTGCAAGGAATGGTTCAGAATGTGACGGGGCTCGAAGCTCTTGCTGATGTCGAAGATCTTACTGTGACGTATGGCATCGTGACGAATTTTCTC
   PhCRN_07-r304 GAGAATTTCACTTGTCGAAGCAAAGCGTGAAGATATGCTGCAAGGAATGGTTCAGAATGTGACGGGGCTCGAAGCTCTTGCTGATGTCGAAGATCTTACTGTGACGTATGGCATTGTGACGAATTTTCTC
   PhCRN_07-r100 GAGAATTTCACTTGTCGAAGCAAAGCGTGAAGATATGCTGCAAGGAATGGTTCAGAATGTGACGGGGCTCGAAGCTCTTGCTGATGTCGAAGATCTTACTGTGACGTATGGCATTGTGACGAATTTTCTC
   PhCRN_07-r703 GAGAATTTCACTTGTCGAAGCAAAGCGTGAAGATATGCTGCAAGGAATGGTTCAGAATGTGACGGGGCTCGAAGCTCTTGCTGATGTCGAAGATCTTACTGTGACGTATGGCATTGTGACGAATTTTCTC
       Consensus GAGAATTTCACTTGTCGAAGCAAAGCGTGAAGATATGCTGCAAGGAATGGTTCAGAATGTGACGGGGCTCGAAGCTCTTGCTGATGTCGAAGATCTTACTGTGACGTATGGCATtGTGACGAATTTTCTC

                1041     1050      1060      1070      1080      1090      1100      1110      1120      1130      1140      1150      1160      1170
                |--------+---------+---------+---------+---------+---------+---------+---------+---------+---------+---------+---------+---------|
       PhCRN_07 GAGTGGAAGTTCTTGATCAGTGGCGACGAGAAGGTCAGAGAGCATGAGACTGTTCTTCCTCAAGCGAATACCATACCGTCGTTTGAAGGATTGAAAGAAATTGTGGGGAAAATTTACGCGATGCTGCAAT
   PhCRN_07-r710 GAGTGGAAGTTCTTGATCAGTGGCGACGAGAAGGTCAGAGAGCATGAGACTGTTCTTCCTCAAGCGAATACCATACCGTCGTTTGAAGGATTGAAAGAAATTGTGGGGAAAATTTACGCGATGCTGCAAT
   PhCRN_07-r304 GAGTGGAAGTTCTTGATCAGTGGCGACGAGAAGGTCAGAGAGCATGAGACTGTTCTTCCTCAAGCGAATACCATACCGTCGTTTGAAGGATTGAAAGAAATTGTGGGGAAAATTTACGCGATGCTGCAAT
   PhCRN_07-r100 GAGTGGAAGTTCTTGATCAGTGGCGACGAGAAGGTCAGAGAGCATGAGACTGTTCTTCCTCAAGCGAATACCATACCGTCGTTTGAAGGATTGAAAGAAATTGTGGGGAAAATTTACGCGATGCTGCAAT
   PhCRN_07-r703 GAGTGGAAGTTCTTGATCAGTGGCGACGAGAAGGTCAGAGAGCATGAGACTGTTCTTCCTCAAGCGAATACCATACCGTCGTTTGAAGGATTGAAAGAAATTGTGGGGAAAATTTACGCGATGCTGCAAT
       Consensus GAGTGGAAGTTCTTGATCAGTGGCGACGAGAAGGTCAGAGAGCATGAGACTGTTCTTCCTCAAGCGAATACCATACCGTCGTTTGAAGGATTGAAAGAAATTGTGGGGAAAATTTACGCGATGCTGCAAT

                1171     1180      1190      1200      1210      1220      1230      1240      1250      1260      1270      1280      1290      1300
                |--------+---------+---------+---------+---------+---------+---------+---------+---------+---------+---------+---------+---------|
       PhCRN_07 AAATATCTTCCATATGAGTTGCAATCCTTTTGCTTATTTTCTAATTGAGAT-CTTCTTACATGCACCCATCTGCTTAATCATGGCATCGAAAGGTATCTCTGATCGTCCGCTCAACGATAGCACTTAAGA
   PhCRN_07-r710 AAATATCTTCCATATGAGTTGCAATCCTTTTGCTTATTTTCTAATTGAGAT-CTTCTTACATGCACCCATCTGCTTAATCATGGCATCGAAAGGTATCTCTGATCGTCCGCTCAACGATAGCACTTAAGA
   PhCRN_07-r304 AAATATCTTCCATATGAGTTGCAATCCTTTTGCTTATTTTCTAATTGAGATTCTTCTTACAT
   PhCRN_07-r100 AAATATCTTCCATATGAGTTGCAATCCTTTTGCTTATTTTCTAATTGAGAT-CTTCTTACATGCACCCATCTGCTTAATCATGGCATCGAAAGGTATCTCTGATCGTCCGCTCAACGATAGCACTTAAGA
   PhCRN_07-r703 AAATATCTTCCATATGAGTTGCAATCCTTTTGCTTATTTTCTAATTGAGA
       Consensus AAATATCTTCCATATGAGTTGCAATCCTTTTGCTTATTTTCTAATTGAGAt.cttcttacatgcacccatctgcttaatcatggcatcgaaaggtatctctgatcgtccgctcaacgatagcacttaaga
```

PhCRN_07_AA

```
                    131      140       150       160       170       180       190       200       210       220       230       240       250       260
                    |--------+---------+---------+---------+---------+---------+---------+---------+---------+---------+---------+---------+---------|
PhCNR_07-cluster ENDRFAAMAIGVAPSLPPTTIHRHPERLKRWAAINAMIRQKNQEANQKTTSEDAKKTNKKRKNHDVDKTVGCSSLCWEDIKPIYHFDDSFDLQPSDIPDADIELLLARIRDLRELYGQISDGKEAKRLFF
   PhCRN_07-r304 ENDRFAAMAIGVAPSLPPTTIHRHPERLKRWAAINAMIRQKNQEANQKTTSEDAKKTNKKRKNHDVDKTVGCSSLCWEDIKPIYHFDDSFDLQPSDIPDADIELLLARIRDLRELYGQISDGKEAKRLFF
   PhCRN_07-r710 ENDRFAAMAIGVAPSLPPTTIHRHPERLKRWAAINAMIRQKNQEANQKTTSEDAKKTSKKRKNHDVDKTVGCSSLCWEDIKPIYHFDDSFDLQPSDIPDADIELLLARIRDLRELYGQISDGKEAKRLFF
   PhCRN_07-r100 ENDRFAAMAIGVAPSLPPTTIHRHPERLKRWAAINAMIRQKNQEANQKTTSEDAKKTNKKRKNHDVDKTVGCSSLCWEDIKPIYHFDDSFDLQPSDIPDADIELLLARIRDLRELYGQISDGKEAKRLFF
   PhCRN_07-r703                                                   TTSEDAKKTSKKRKNHDVDKTVGCSSLCWEDIKPIYHFDDSFDLQPSDIPDADIELLLARIRDLRELYGQISDGKEAKRWFF
       Consensus endrfaamaigvapslpptti hrhperlkrwaainamirqknqeanqkTTSEDAKKTsKKRKNHDVDKTVGCSSLCWEDIKPIYHFDDSFDLQPSDIPDADIELLLARIRDLRELYGQISDGKEAKRlFF

                    261      270       280       290       300       310       320       330       340       350       360       370       380      389
                    |--------+---------+---------+---------+---------+---------+---------+---------+---------+---------+---------+---------+--------|
PhCNR_07-cluster IAPILETVSRLLKNVRILVEEDVVGKNVLLKGRFEFVLKRGTKRISLVEAKREDMLQGMVQNVTGLEALADVEDLTVTYGIVTNFLEWKFLISGDEKVREHETVLPQANTIPSFEGLKEIVGKIYAMLQ
   PhCRN_07-r304 IAPILETVSRLLKNVRILVEEDVVGKNVLLKGRFEFVLKRGTKRISLVEAKREDMLQGMVQNVTGLEALADVEDLTVTYGIVTNFLEWKFLISGDEKVREHETVLPQANTIPSFEGLKEIVGKIYAMLQ
   PhCRN_07-r710 IAPILETVSRLLKNVRILVEEDVVGKNVLLKGRFEFVLKRGTKRISLVEAKREDMLQGMVQNVTGLEALADVEDLTVTYGIVTNFLEWKFLISGDEKVREHETVLPQANTIPSFEGLKEIVGKIYAMLQ
   PhCRN_07-r100 IAPILETVSRLLKNVRILVEEDVVGKNVLLKGRFEFVLKRGTKRISLVEAKREDMLQGMVQNVTGLEALADVEDLTVTYGIVTNFLEWKFLISGDEKVREHETVLPQANTIPSFEGLKEIVGKIYAMLQ
   PhCRN_07-r703 IAPILETVSRLLKNVRILVEEDVVGKNVLLKGRFEFVLKRGTKRISLVEAKREDMLQGMVQNVTGLEALADVEDLTVTYGIVTNFLEWKFLISGDEKVREHETVLPQANTIPSFEGLKEIVGKIYAMLQ
       Consensus IAPILETVSRLLKNVRILVEEDVVGKNVLLKGRFEFVLKRGTKRISLVEAKREDMLQGMVQNVTGLEALADVEDLTVTYGIVTNFLEWKFLISGDEKVREHETVLPQANTIPSFEGLKEIVGKIYAMLQ
```

PhCRN_09_NA

```
                   781     790       800       810       820       830       840       850       860       870       880       890       900       910
                   |--------+---------+---------+---------+---------+---------+---------+---------+---------+---------+---------+---------+---------|
PhCRN_09_cluster   GCGTCTCATCATTGGTTGAATATCTATGTCACACGCCTTCTTTTCCGCTTAAGTACGCCAGAAATTCTCCCTCCCTTCTTCCCTCTGATTCCAGAATCTGCCGGCTCGAGGAAAGATGGTTGAAATCTCA
PhCRN_09-r703_ctg2 GCGTCTCATCATTGGTTGAATATCTATGTCACACGCCTTCTTTTCCGCTTAAGTACGCCAGAAATTCTCCCTCCCTTCTTCC---TCATTCCAGAATCTGCCGGCTCGAGGAAAGATGGTTGAAATCTCA
PhCRN_09-r710      ---------------------------------------------------------------------------------------ATTCCAGAATCTGCCGGCTCGAGGAAAGATGGTTGAAATCTCA
PhCRN_09-r304      --------------------------------------------------------------------------------------GATTCCAGAATCTGCCGGCTCGAGGAAAGATGGTTGAAATCTCA
Consensus          ......................................................................................gATTCCAGAATCTGCCGGCTCGAGGAAAGATGGTTGAAATCTCA

                   911     920       930       940       950       960       970       980       990       1000      1010      1020      1030      1040
                   |--------+---------+---------+---------+---------+---------+---------+---------+---------+---------+---------+---------+---------|
PhCRN_09_cluster   CTGGAGTGTGCAATCGAAGGCCAGGTAGGCTCGTTCAACGTGACGATCGACGACGGCAAGAAGATTTCTGTGTTGAAGGACGCGATCAAGGATAAGAAGCCGAACAAGTTGAAGAACGTTGATGCCGACG
PhCRN_09-r703_ctg2 CTGGAGTGTGCAATCGAAGGCCAGGTAGGCTCGTTCAACGTGACGATCGACGACGGCAAGAAGATTTCTGTGTTGAAGGACGCGATCAAGGATAAGAAGCCGAACAAGTTGAAGAACGTTGATGCCGACG
PhCRN_09-r710      CTGGAGTGTGCAATCGAAGGCCAGGTAGGCTCTTTCAACGTGACGATCGACGACGGCAAGAAGATTTCTGTGTTGAAGGACGCGATCAAGGATAAGAAGCCGAACAAGTTGAAGAACGTTGATGCCGACG
PhCRN_09-r304      CTGGAGTGTGCAATCGAAGGCCAGGTAGGCTCGTTCAACGTGACGATCGACGACGGCAAGAAGATTTCTGTGTTGAAGGACGCGATCAAGGATAAGAAGCCGAACAAGTTGAAGAACGTTGATGCCGACG
Consensus          CTGGAGTGTGCAATCGAAGGCCAGGTAGGCTCgTTCAACGTGACGATCGACGACGGCAAGAAGATTTCTGTGTTGAAGGACGCGATCAAGGATAAGAAGCCGAACAAGTTGAAGAACGTTGATGCCGACG

                   1041    1050      1060      1070      1080      1090      1100      1110      1120      1130      1140      1150      1160      1170
                   |--------+---------+---------+---------+---------+---------+---------+---------+---------+---------+---------+---------+---------|
PhCRN_09_cluster   AGCTGGAGCTCTCTCTGGCGAAGAAGGGCGAAGATTGGCTACCAATTGAGGACCTTCGTGCGATACGAAAAGGAGAGGATGTACCGGGGTTCGAAAGGGTATCACTGGTGGATACAGATGATGAAGAGTA
PhCRN_09-r703_ctg2 AGCTGGAGCTCTCTCTGGCGAAGAAGGGCGAAGATTGGCTACCAATTGAGGACCTTCGTGCGATACGAAAAGGAGAGGATGTACCGGGGTTCGAAAGGGTATCACTGGTGGATACAGATGATGAAGAGTA
PhCRN_09-r710      AGCTGGAGCTCTCTCTGGCGAAGAAGGGCGAAGATTGGCTACCAATTGAGGACCTTCGTGCGATACGAAAAGGAGAGGATGTACCGGGGTTCGAAAGGGTATCACTGGTGGATACAGATGATGAAGAGTA
PhCRN_09-r304      AGCTGGAGCTCTCTCTGGCGAAGAAGGGCGAAGATTGGCTACCGATTGAGGACCTTCGTGCGATACGAAAAGGAGAGGATGTACCGGGGTTCGAAAGGGTATCACTGGTGGATACAGATGATGAAGAGTA
Consensus          AGCTGGAGCTCTCTCTGGCGAAGAAGGGCGAAGATTGGCTACCaATTGAGGACCTTCGTGCGATACGAAAAGGAGAGGATGTACCGGGGTTCGAAAGGGTATCACTGGTGGATACAGATGATGAAGAGTA

                   1171    1180      1190      1200      1210      1220      1230      1240      1250      1260      1270      1280      1290      1300
                   |--------+---------+---------+---------+---------+---------+---------+---------+---------+---------+---------+---------+---------|
PhCRN_09_cluster   CTCGGCCTATTCGATTCAGAAAATGTTGCAGATGAAAGGATTGCGCTCACCTCAAACGGAGCAAATTCACGTGCTGGTGGTGGTTCCTCCGCCGAGTGTCGGGTCGAAGCGTTCAGCTGATGAAATTGCT
PhCRN_09-r703_ctg2 CTCGG
PhCRN_09-r710      CTCGGCCTATTCGATTCAGAAAATGTTGCAGATGAAAGGATTGCGCTCACCTCAAACGGAGCAAATTCACGTGCTGGTGGTGGTTCCTCCGCCGAGTGTCGGGTCGAAGCGTTCAGCTGATGAAATTGCT
PhCRN_09-r304      CTCGGCCTATTCGATTCAGAAAATGTTGCAGATGAAAGGATTGCGCTCACCTCAA-CGGAGCAAATTCACGTGCTGGTGGTGGTTCCTCCGCCGAGTGTCGG-TCGAAGCGTTCAGCTGATG
Consensus          CTCGGcctattcgattcagaaaatgttgcagatgaaaggattgcgctcacctcaa.cggagcaaattcacgtgctggtggtggttcctccgccgagtgtcgg.tcgaagcgttcagctgatg........
```

PhCRN_09_AA

```
                   1       10        20        30        40        50        60        70        80        90        100       110       120       130
                   |--------+---------+---------+---------+---------+---------+---------+---------+---------+---------+---------+---------+---------|
PhCRN_09_cluster   MVEISLECAIEGQVGSFNVTIDDGKKISVLKDAIKDKKPNKLKNVDADELELSLAKKGEDWLPIEDLRAIRKGEDVPGFERVSLVDTDDEEYSAYSIQKMLQMKGLRSPQTEQIHVLVVVPPPSVGSKRS
PhCRN_09-r710      MVEISLECAIEGQVGSFNVTIDDGKKISVLKDAIKDKKPNKLKNVDADELELSLAKKGEDWLPIEDLRAIRKGEDVPGFERVSLVDTDDEEYSAYSIQKMLQMKGLRSPQTEQIHVLVVVPPPSVGSKRS
PhCRN_09-r304      MVEISLECAIEGQVGSFNVTIDDGKKISVLKDAIKDKKPNKLKNVDADELELSLAKKGEDWLPIEDLRAIRKGEDVPGFERVSLVDTDDEEYSAYSIQKMLQMKGLRSPQTEQIH
PhCRN_09-r703_c2   MVEISLECAIEGQVGSFNVTIDDGKKISVLKDAIKDKKPNKLKNVDADELELSLAKKGEDWLPIEDLRAIRKGEDVPGFERVSLVDTDDEEYS
Consensus          MVEISLECAIEGQVGSFNVTIDDGKKISVLKDAIKDKKPNKLKNVDADELELSLAKKGEDWLPIEDLRAIRKGEDVPGFERVSLVDTDDEEYSaysiqkmlqmkglrspqteqih...............

                   131     140       150       160       170       180    187
                   |--------+---------+---------+---------+---------+------|
PhCRN_09_cluster   ADEIADVQKRLRLLETDSAAIKPALAAIKPKTLTKFTPLSVHEDEFQLDSLSITQQK
PhCRN_09-r710      ADEIADVQKRLRLLETDS
PhCRN_09-r304
PhCRN_09-r703_c2
Consensus          .........................................................
```
